# Macrophage OTUD1‐CARD9 axis drives isoproterenol‐induced inflammatory heart remodelling

**DOI:** 10.1002/ctm2.1790

**Published:** 2024-08-08

**Authors:** Jinfu Qian, Qinyan Wang, Jiachen Xu, Shiqi Liang, Qingsong Zheng, Xiaocheng Guo, Wu Luo, Weijian Huang, Xiaohong Long, Julian Min, Yi Wang, Gaojun Wu, Guang Liang

**Affiliations:** ^1^ Department of Cardiology the First Affiliated Hospital of Wenzhou Medical University Wenzhou China; ^2^ Chemical Biology Research Center School of Pharmaceutical Sciences Wenzhou Medical University Wenzhou China; ^3^ School of Pharmaceutical Sciences Hangzhou Medical College Hangzhou China

**Keywords:** CARD9, heart failure, inflammation, isoproterenol, macrophage, OTUD1

## Abstract

**Background:**

Chronic inflammation contributes to the progression of isoproterenol (ISO)‐induced heart failure (HF). Caspase‐associated recruitment domain (CARD) families are crucial proteins for initiation of inflammation in innate immunity. Nonetheless, the relevance of CARDs in ISO‐driven cardiac remodelling is little explored.

**Methods:**

This study utilized Card9^−/−^ mice and reconstituted C57BL/6 mice with either Card9^−/−^ or Otud1^−/−^ marrow‐derived cells. Mechanistic studies were conducted in primary macrophages, cardiomyocytes, fibroblasts and HEK‐293T cells.

**Results:**

Here, we demonstrated that CARD9 was substantially upregulated in murine hearts infused with ISO. Either whole‐body CARD9 knockout or myeloid‐specific CARD9 deletion inhibited ISO‐driven murine cardiac inflammation, remodelling and dysfunction. CARD9 deficiency in macrophages prevented ISO‐induced inflammation and alleviated remodelling changes in cardiomyocytes and fibroblasts. Mechanistically, we found that ISO enhances the activity of CARD9 by upregulating ovarian tumour deubiquitinase 1 (OTUD1) in macrophages. We further demonstrated that OTUD1 directly binds to the CARD9 and then removes the K33‐linked ubiquitin from CARD9 to promote the assembly of the CARD9‐BCL10‐MALT1 (CBM) complex, without affecting CARD9 stability. The ISO‐activated CBM complex results in NF‐κB activation and macrophage‐based inflammatory gene overproduction, which then enhances cardiomyocyte hypertrophy and fibroblast fibrosis, respectively. Myeloid‐specific OTUD1 deletion also attenuated ISO‐induced murine cardiac inflammation and remodelling.

**Conclusions:**

These results suggested that the OTUD1‐CARD9 axis is a new pro‐inflammatory signal in ISO‐challenged macrophages and targeting this axis has a protective effect against ISO‐induced HF.

**Key points:**

- Macrophage CARD9 was elevated in heart tissues of mice under chronic ISO administration.
- Either whole‐body CARD9 knockout or myeloid‐specific CARD9 deficiency protected mice from ISO‐induced inflammatory heart remodeling.
- ISO promoted the assembly of CBM complex and then activated NF‐κB signaling in macrophages through OTUD1‐mediated deubiquitinating modification.
- OTUD1 deletion in myeloid cells protected hearts from ISO‐induced injuries in mice.

## Graphical abstract

- Macrophage CARD9 was elevated in the heart tissues of mice under chronic ISO administration.
- Either whole‐body CARD9 knockout or myeloid‐specific CARD9 deficiency protected mice from ISO‐induced inflammatory heart remodeling.
- ISO promoted the assembly of the CBM complex and then activated NF‐κB signalling in macrophages through OTUD1‐mediated deubiquitinating modification.
- OTUD1 deletion in myeloid cells protected hearts from ISO‐induced injuries in mice.

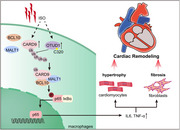

## 1 INTRODUCTION

Extracellular stress, including neurohumoral mediators and biomechanical stress, activates intracellular signalling pathways in the heart, finally inducing cardiac hypertrophy. Interestingly, the initial adaptive response is beneficial in that it facilitates maintenance of normal cardiac activity, however, continued pathological hypertrophy can eventually result in heart failure (HF).^1^ High‐dose isoproterenol (ISO), a sympathomimetic nonselective β‐adrenergic receptor (β‐AR) agonist, is reported to induce myocardial inflammation, necrosis, hypertrophy, fibroblast proliferation and aberrant systolic‐diastolic activity, which mimics the pathological alterations that occur in human HF cardiac tissue.^2^ Recent evidences showed that the elevation of infiltrated macrophages and proinflammatory mediators is observed in ISO‐induced cardiac remodeling.^3^, ^4^ Prolonged unchallenged inflammation eventually gives rise to HF development and progression,^5^ indicating that cardiac inflammation is a potential target for innovative treatment of HF.^6^, ^7^ Despite the availability of several effective therapies for HF, its morbidity and mortality rates remain high, imposing significant burdens on society.^8^ Hence, identification of novel molecules or modulatory signalling networks related to cardiac inflammation is critically necessary to prevent and/or treat adrenergic stimulation‐related HF.

Caspase‐associated recruitment domain (CARD)‐containing sensor and adaptor protein families are essential for initiation of inflammation in innate immunity response to microbial infection.^9^ Recent studies also showed that the intracellular CARD family of proteins mediates downstream signalling in sterile inflammation of certain non‐infectious disorders, such as heart diseases.^10^, ^11^ Recent research has shown that RIPK2 (CARD3), NOD1 (CARD4), CARD6, CARD9, NOD2 (CARD15) play protective roles in hearts against hypertrophy caused by pressure overload.^12^, ^13^, ^14^, ^15^, ^16^ However, the involvement of CARD family member in cardiac remodelling induced by ISO remains largely has not been extensively studied. Herein, we found that, among the CARD family, CARD9 was most highly expressed in mouse heart tissues under ISO infusion. CARD9 is expressed particularly in immunoreactive cells, such as macrophages, neutrophils and dendritic cells. Emerging evidence demonstrated that CARD9 could interact with B‐cell lymphoma/leukaemia 10 (BCL10) and mucosa‐associated lymphoid tissue lymphoma translocation protein 1 (MALT1) to generate a CARD9‐BCL10‐MALT1 complex (CBM) to regulate the production of cytokines and chemokines via activating nuclear factor κB (NF‐κB) in presence of extra‐ and intracellular proinflammatory stimuli.^17^ Although the role of CARD9 in HF remains unclear, we consider that exploring the function of CARD9 and its underlying mechanisms in ISO‐induced HF may reveal new targets or strategies for treating this disease.

In this study, we further found that myeloid‐specific CARD9 deficiency protected mice from ISO‐driven cardiac inflammation, remodelling and dysfunction. Mechanistically, we demonstrated that a deubiquitinating enzyme, ovarian tumour deubiquitinase 1 (OTUD1), directly deubiquitinated CARD9 to promote the assembly of the CBM complex and then activate NF‐κB signalling in macrophages. This paper identified the OTUD1‐CARD9 axis in macrophages as a newly identified modulator of ISO‐stimulated cardiac remodelling.

## 2 METHODS AND MATERIALS

### 2.1 General reagents

The reagents utilized in our experiments are comprehensively listed in Table S1.

### 2.2 Animal experiments

Male C57BL/6 (wild‐type, WT) mice were sourced from the Laboratory Animal Center of Wenzhou Medical University. Card9 knockout mice were generously by Prof. Xinming Jia from Tongji University School of Medicine. Otud1 knockout mice were obtained from Prof. You Fuping from Peking University Health Science Center. All animals were kept under constant room temperature and a 12 h light/dark cycle with lights turning on at 7:30 AM. The timing of our animal experiments is concentrated in the morning and all experiments of different groups were conducted at a consistent time to minimize variability possibly caused by the circadian rhythms or other time‐related factors. All mice protocols received approval by the Laboratory Animal Ethics Committee of Wenzhou Medical University (approval no. wydw2021‐0438) and abided by the guidelines established by the National Institutes of Health.

Model 1: 7−8 weeks old male Card9^−/−^ and WT mice were arbitrarily separated into four cohorts (*n* = 6): WT + Sham, WT + ISO, Card9^−/−^ + Sham and Card9^−/−^ + ISO. 30 mg·kg^−1^·day^−1^ ISO was given via osmotic pumps over 2 weeks, as reported in a prior article.^18^

Model 2: Bone marrow transplantation (BMT) model 1. One week before transplantation, 5−6 weeks old male WT mice were provided with acidified water with neomycin and polymyxin B sulphate. Mice then underwent total body irradiation (800 cGy X‐rays). After 12 h, these WT mice were then intravenously administered with 5 × 10^6^ BM cells from 6 weeks old male Card9^−/−^ or WT mice, as previously described.^18^ After 3 weeks, the chimeric mice received ISO as aforementioned.

Model 3: BMT model 2. One week before transplantation, 5−6 weeks old male WT mice were provided with acidified water with neomycin and polymyxin B sulphate. Mice were subjected to total body irradiation (800 cGy X‐rays). After 12 h, intravenous injection of 5 × 10^6^ BM cells from 6 weeks old male Otud1^−/−^ mice or WT mice to these recipient mice. After 3 week, the chimeric mice were received ISO as aforementioned.

Mice were anaesthetized with 1−2% isoflurane and underwent transthoracic echocardiography (Vevo 3100, Fujifilm VisualSonics) to evaluate cardiac function one day before sacrifice. Following the sacrifice of the mice under isoflurane anaesthesia, heart tissues and blood were extracted for further assessment. RT‐qPCR analysis of primary mouse peritoneal macrophages (MPMs) and heart samples was used to validate the effectiveness of the BMT.

### 2.3 Single‐cell RNA sequencing

Hearts were dissociated into single cells by dissociation solution. Using the 10X Chromium platform (10X Genomics) 3‐prime kit (v2), we built a single‐cell library. The cDNA amplification, library preparation and sequencing were performed by LC‐BIO. The demultiplexing, barcoded processing, gene counting and aggregation were done using the Cellranger v6.0. Seurat v4.0 was applied to perform the subsequent analysis. The data have been uploaded to the GEO database (GSE271946).

### 2.4 Cell isolation and culture

MPMs isolation utilized a previously reported protocol.^19^ Mice were administered with a single intraperitoneal 6% thioglycollate solution. After 36–48 h, mice were euthanized and their peritoneal cavity was perfused with RPMI‐1640 medium. The harvested cells were quantified and seeded in RPMI‐1640 medium supplemented with 10% FBS, 100 mg/mL streptomycin and 100 U/mL penicillin.

The isolation and culture of adult mouse cardiomyocytes (ACMs) and fibroblasts (ACFs) were followed by previously established methods.^20^ The digestion process consisted of sequential injections into the left ventricle administered with a peristaltic pump: EDTA buffer, perfusion buffer (EDTA‐free) and collagenase buffer (0.05 mg/mL Protease XIV, 0.5 mg/mL Collagenase II and Collagenase IV,). The cardiac myocytes were allowed to settle through four gravity sedimentation steps, interspersed with three intermediate buffers that incrementally reintroduced calcium to physiologic levels. Cardiac fibroblasts were harvested from the supernatant after the first settling phase of the cardiomyocytes and cultured, passaged and then collected. Cardiomyocytes and fibroblasts were grown in DMEM with 4.5 g/L glucose, 10% FBS, 100 U/mL penicillin and 100 mg/mL streptomycin.

The isolation of cardiomyocytes, fibroblasts and macrophages was performed under strictly standardized conditions. For each group, cells were isolated using the same protocol and by the same technician to ensure consistency within all treatments and comparisons. In addition, the HEK 293T cells were acquired from the Cell Bank, Chinese Academy of Sciences, and grown in the same culture hub as the ACMs.

### 2.5 RT‐qPCR

We extracted and purified total RNA from tissues and cells using RNAiso Plus. The isolated RNA was subsequently reverse‐transcribed into cDNA using the PrimeScript RT reagent kit. For RT‐qPCR analysis, we used the SYBR Green reagent kit. The primers used for this analysis, obtained from Tsingke, are summarized in Table S2.

### 2.6 Western blotting and co‐immunoprecipitation

The protein isolation from heart tissues and cells was conducted using RIPA buffer. Protein quantification was done via the Bradford assay. Equal quantities of proteins were electrophoresed and transferred to PVDF membranes. The membranes were blocked using 5% non‐fat dry milk, followed by incubation with primary antibodies, and subsequently with secondary antibodies. The immunolabelled proteins were detected via enhanced chemiluminescence, and protein quantification utilized ImageJ.

Using co‐immunoprecipitation and immunoblotting (IB), we obtained protein‐bead complexes. The cell lysates were incubated with primary antibodies, and subsequently with protein A+G‐sepharose beads. Protein‐bead complexes were rinsed, denaturated, electrophoresed, transferred and identified using an IB antibody. In addition, immune complexes were detected the same as in western blotting. Target protein quantification utilized ImageJ.

### 2.7 Immunofluorescence staining

Frozen tissues underwent embedding in OCT compound and were sectioned into 5 µm thick slices for CARD9, F4/80, α‐actinin and Vimentin staining. Slides were counterstained with DAPI. MPMs were stained with NF‐κB P65. Staining observation image capture utilized the fluorescence microscope (Nikon).

### 2.8 Histopathological evaluation

The partial tissue underwent fixation in 4% formaldehyde, followed by dehydration, embedding in paraffin and sectioned into 5 µm thick slices, with subsequent H&E staining for histopathological assessment and Sirius Red staining to evaluate cardiac fibrosis under the light microscope (Nikon).

### 2.9 Enzyme‐linked immunosorbent assay

IL‐6 and TNF‐α contents were assessed via corresponding enzyme‐linked immunosorbent assay kits and associated protocols.

### 2.10 Gene overexpression

Gene overexpression was established in cells by transfecting unique plasmids. Plasmids encoding Flag‐OTUD1, Flag‐OTUD1 C320S, HA‐Ub, HA‐Ub‐K33 were generated by Genechem (Shanghai). Plasmid encoding Myc‐CARD9 and GFP‐BCL10 were constructed by Sino Biological (Beijing). Transfection of 293T cells and MPMs with plasmids was conducted via Lipofectamine 3000.

### 2.11 Statistical analysis

All experiments were random and blinded. The data are presented as the mean ± SEM. Data normality was assessed using the Shapiro–Wilk test. For comparison between the two groups, a two‐tailed Student's *t*‐test was used for normally distributed data, while the Mann–Whitney *U* test was applied for non‐normal distribution data. Multiple group comparisons were performed with one‐way ANOVA followed by Tukey's multiple comparison test when the assumption of normal distribution was satisfied. Otherwise, the Kruskal–Wallis test was used for further analysis. Two‐way ANOVA with Tukey's multiple comparison test was employed when analyzing interactions between more than two variables. *p* < 0.05 was considered significant.

## 3 RESULTS

### 3.1 Macrophage CARD9 was elevated in heart tissues of mice under chronic ISO administration

**FIGURE 1:**
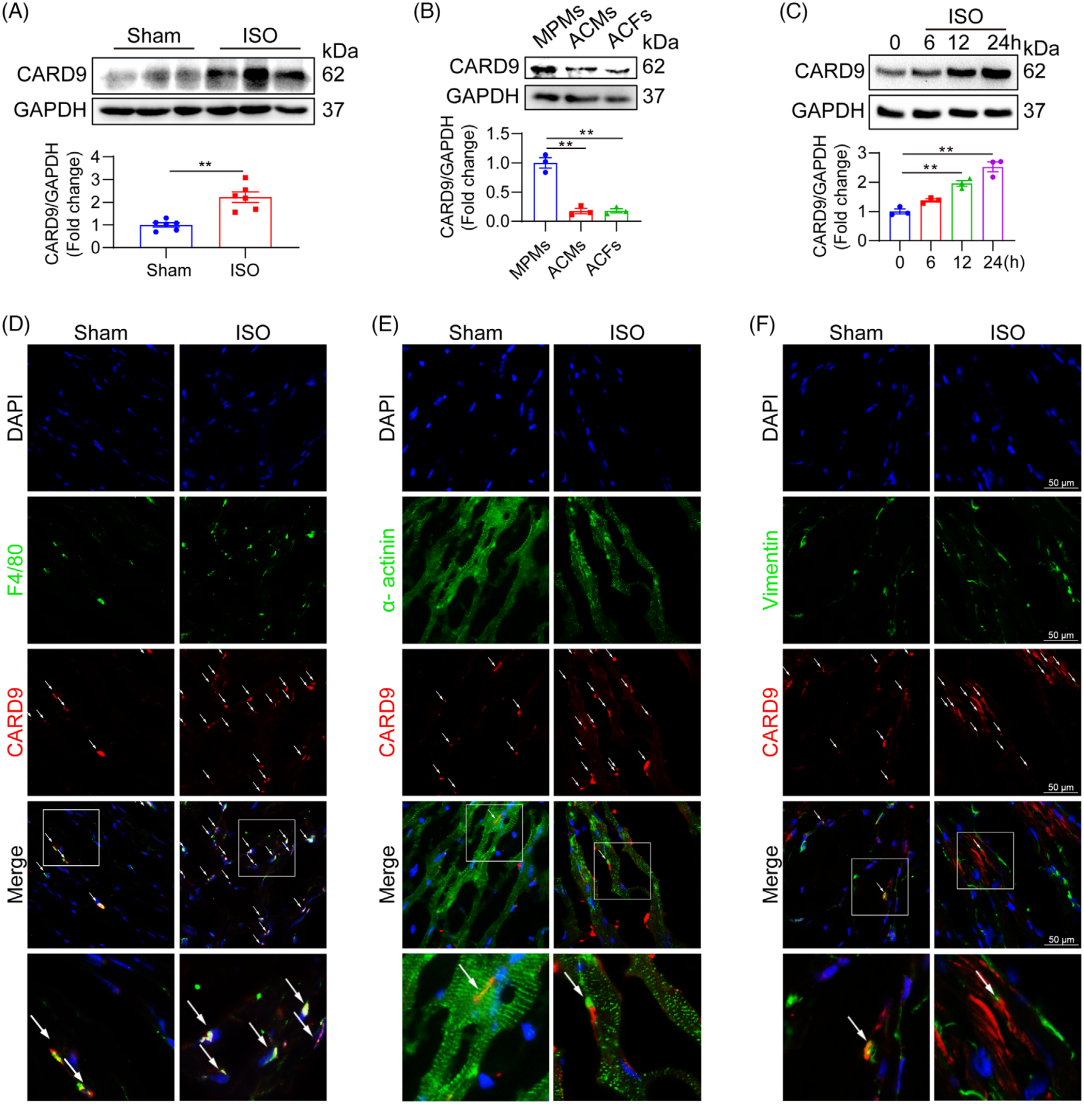
Macrophage CARD9 was elevated in heart tissues under chronic ISO administration in mice. (A). WT mice were provided with 30 mg·kg^−1^·day^−1^ ISO over a period of two weeks. Typical immunoblots of CARD9 in cardiac tissues. GAPDH served as the loading reference (*n* = 6). (B) Representative immunoblots of CARD9 in MPMs, ACMs and ACFs. GAPDH was the loading reference (*n* = 3). (C) MPMs were exposed to 20 µM ISO for specific durations. CARD9 protein expressions were examined through Western blotting analysis (*n* = 3). (D–F) WT mice were provided with 30 mg·kg^−1^·day^−1^ ISO over 2 weeks. Typical immunofluorescence images depicting CARD9 (red) and F4/80 (green, D), α‐actinin (green, E) or Vimentin (green, F) staining in heart sections with ISO infusion. Sections received DAPI counterstaining (blue). Arrows indicate CARD9‐ positive cells (scale bar = 50 µm). Data provided as mean ± SEM; **p < 0.01.

We determined whether Card family genes are linked to murine ISO‐stimulated cardiac injury. We demonstrated a marked rise in the Card6, Card9, Card11, Card14 and Card15 transcript expressions in mouse cardiac tissues following ISO infusion. Among them, the Card9 gene exhibited the largest changing fold (Figure S1). We also noted an upregulation in CARD9 protein expression in ISO‐challenged mouse hearts (Figure 1A). Our single‐cell RNA sequencing data using the mouse cardiac tissues of the Sham group showed that the Card9 mRNA expression was mainly distributed in macrophages (Figure S2). To elucidate the cellular source of enhanced CARD9 in ISO‐driven models, we analyzed CARD9 protein levels in cultured MPMs, ACMs and ACFs. CARD9 was predominantly expressed in macrophages and showed a time‐dependent increase upon ISO challenge (Figure 1B,C). The double immunofluorescence staining also showed that CARD9 expression was increased predominantly in F4/80+ infiltrated macrophages (Figure 1D), rather than α‐actinin+ cardiomyocytes (Figure 1E) and Vimentin+ fibroblasts (Figure 1F). Thus, we identified macrophage CARD9 as a potential regulator in ISO‐based cardiac remodelling.

### 3.2 CARD9 depletion inhibited ISO‐driven cardiac hypertrophy, fibrosis and inflammatory response

**FIGURE 2:**
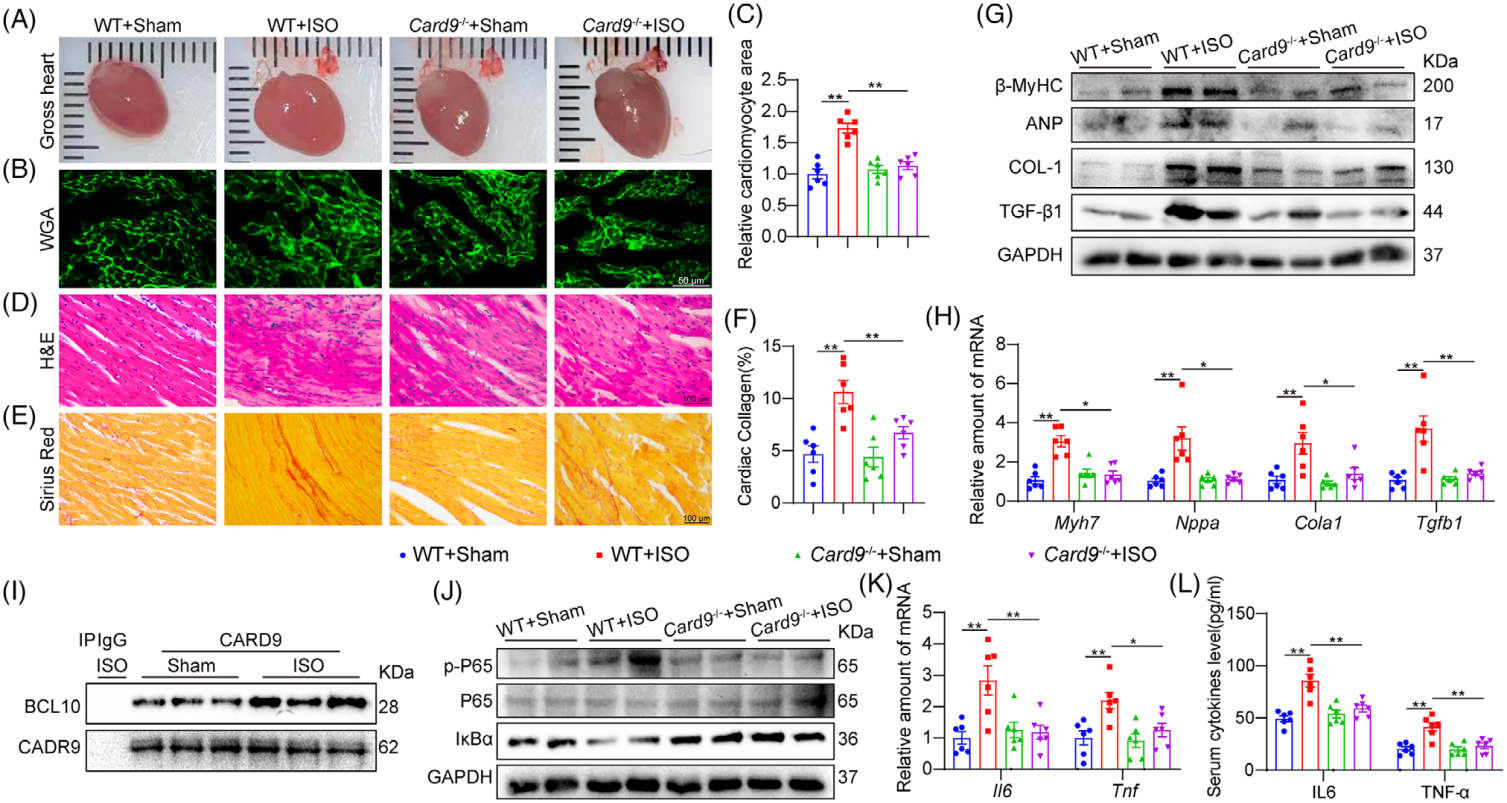
CARD9 deficiency suppressed ISO‐driven cardiac hypertrophy, fibrosis and inflammatory response. Card9^−/−^ and WT mice were provided with 30 mg·kg^−1^·day^−1^ ISO over 2 week. (A) Representative images of gross hearts. (B, C) Cardiomyocyte areas were assessed through WGA staining (scale bar = 50 µm) (B) and quantitative assessment (C) (*n* = 6). (D) Representative H&E staining of cardiac sections (scale bar = 100 µm). (E, F) Typical Sirius Red cardiac sections staining (scale bar = 100 µm) (E) and quantitative analysis (F) (*n* = 6). (G) Representative immunoblots of β‐MyHC, ANP, COL‐1 and TGF‐β1 in cardiac tissue. GAPDH was the loading reference. (H) Myh7, Nppa, Cola1 and Tgfb1 transcript expressions in cardiac tissues were measured by real‐time PCR. Actb mRNA was the loading reference (*n* = 6). (I) Typical immunoblots depicting the CARD9 and BCL10 co‐immunoprecipitation (co‐IP) in cardiac tissues. Endogenous CARD9 was IP using an anti‐CARD9 antibody. IgG served as the IP reference. (J) Typical p‐P65 and IκB‐α protein analyses via western blot assay in mouse cardiac tissue lysates. P65 and GAPDH were the loading references. (K) The cardiac Il6 and Tnf contents as analyzed by RT‐PCR. Actb mRNA was the loading reference (*n* = 6). (L) The circulating IL6 and TNF‐α contents as analyzed by ELISA (*n* = 6). Data presented as mean ± SEM; *p < 0.05; **p < 0.01.

To elucidate the CARD9 significance in ISO‐driven cardiac remodelling, we used Card9^−/−^ mice. Non‐invasive echocardiography showed that CARD9 deficiency attenuated cardiac dysfunction in ISO‐challenged mice, as demonstrated by elevated ejection fraction (EF) and fractional shortening (FS) (Table 1 and Figure S3A). CARD9 deficiency suppressed ISO‐induced hypertrophic response, indicated by decreased gross heart size, heart weight (HW)/body weight (BW), heart weight (HW)/tibia length (TL), left ventricular (LV) mass and LV anterior wall end‐diastolic dimension (Figure 2A and Table 1). WGA staining also revealed that Card9 knockout decreased cardiomyocyte size in ISO‐challenged mice (Figure 2B,C). Based on our histological analysis of cardiac tissues, the level of the structural disorder and interstitial fibrosis were limited by CARD9 insufficiency (Figure 2D–F). Consistently, the protein and mRNA expressions of hypertrophic factors β‐MyHC and ANP, as well as fibrotic factors COL‐1 and TGF‐β1, in ISO‐induced hearts were significantly inhibited by CARD9 knockout (Figure 2G,H and Figure S3B). It is widely reported that CARD9 can recruit and then interact with BCL10 and MALT1 to form a CBM complex that triggers the activation of NF‐κB‐dependent inflammatory response in macrophages.^21^ We examined this signal in heart tissues. The interaction of CARD9 and BCL10 in heart tissues was significantly enhanced under the ISO challenge (Figure 2I and Figure S3C). As a consequence, NF‐κB activation, including P65 phosphorylation and IκB degradation, and inflammatory cytokine overexpression were observed in ISO‐induced mouse hearts, while CARD9 deletion significantly inhibited these changes (Figure 2J–L and Figure S3D). In addition, CARD9 knockout also decreased ISO‐induced the mRNA of chemokines (Cxcr2 and Cxcl1) (Figure S3E). These findings demonstrated that CARD9 depletion abrogates ISO‐driven cardiac dysfunction, remodelling and inflammation.

**TABLE 1:** Echocardiographic demographics of experimental mice.

Model (1)	WT+Sham (*n* = 6)	WT+ISO (*n* = 6)	Card9^−/−^+Sham (*n* = 6)	Card9^−/−^+ISO (*n* = 6)
HW/BW (mg/g)	4.96±0.09	5.87±0.14^**^	5.18±0.13	5.13±0.20^##^
HW/TL (mg/mm)	6.36±0.15	7.99±0.13^**^	6.54±0.18	7.06±0.21^#^
LVIDs (mm)	2.04±0.21	2.56±0.14^ns^	1.90±0.19	2.13±0.19^ns^
LVIDd (mm)	3.15±0.25	3.48±0.15^ns^	2.94±0.25	3.29±0.29^ns^
EF%	66.28±3.05	52.86±1.63^**^	66.73±2.08	65.52±1.69^##^
FS %	36.00±2.23	26.52±0.96^**^	35.67±1.47	35.13±1.32^##^
LV mass (mg)	94.03±10.23	143.6±13.39^*^	78.83±8.02	99.78±11.84^#^
LVAWs (mm)	1.24±0.10	1.42±0.04^ns^	1.23±0.10	1.38±0.07^ns^
LVAWd (mm)	0.74±0.04	1.03±0.03^**^	0.83±0.05	0.88±0.03^#^

*Note*: Transthoracic echocardiography was performed on mice at the end of the animal study.

Abbreviations: BW, body weight; EF, ejection fraction; FS, fractional shortening; HW, heart weight; LVAWd, left ventricular anterior wall end‐diastolic dimension; LVAWs, left ventricular anterior wall end‐systolic dimension; LVIDd, left ventricular end‐diastolic internal diameter; LVIDs, left ventricular end‐systolic internal diameter; ns, not significant; TL, tibial length.

Data presented as mean & SEM, ^*^ *p* < 0.05, ^**^ *p *< 0.01 relative to WT+ Sham. ^#^ *p* < 0.05, ^##^ *p* < 0.01 relative to WT+ ISO.

### 3.3 Marrow‐derived cell CARD9 regulated ISO‐driven cardiac injuries

**FIGURE 3:**
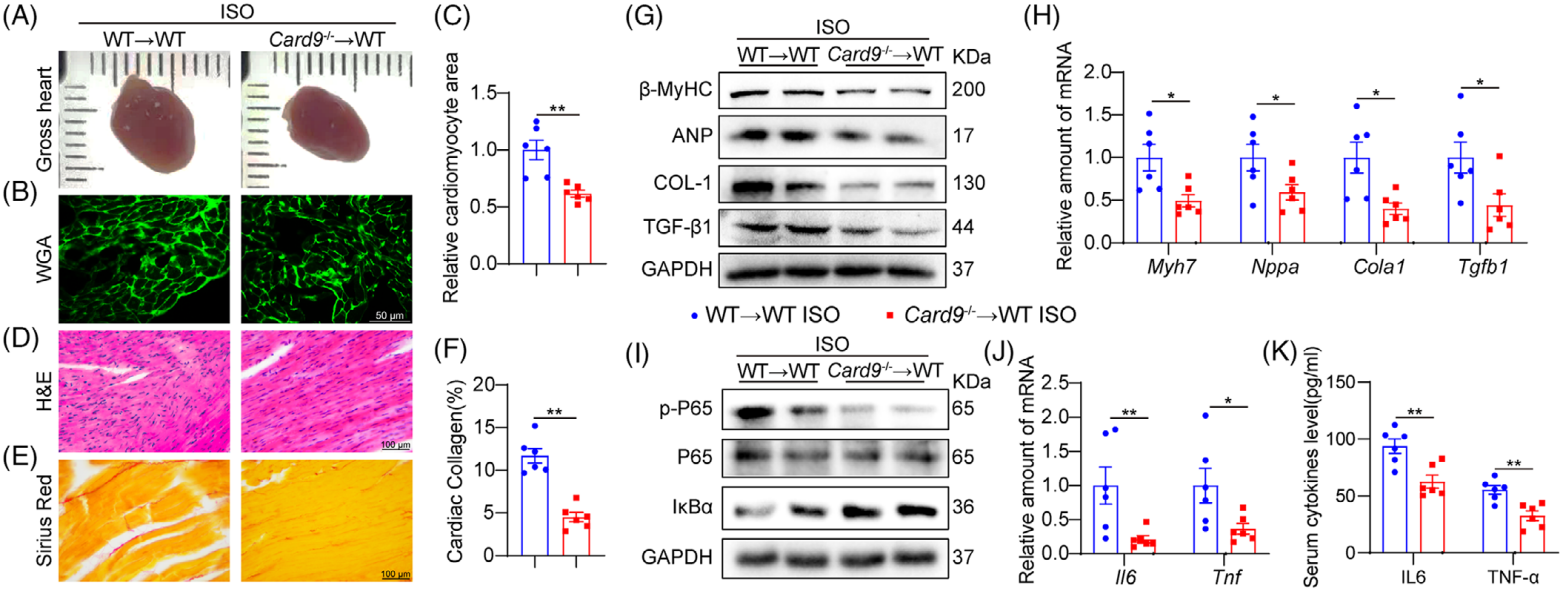
Myeloid cell CARD9 mediated ISO‐driven cardiac injuries. WT mice underwent irradiation and received BMs from either WT or Card9^−/−^ mice. Mice were provided with 30 mg·kg^−1^·day^−1^ ISO over 2 weeks. (A) Representative images of gross hearts. (B,C) Cardiomyocyte areas were assessed through WGA staining (scale bar = 50 µm) (B) and quantitative assessment (C) (*n* = 6). (D) Typical cardiac sections H&E staining (scale bar = 100 µm). (E, F) Typical cardiac sections Sirius Red staining (scale bar = 100 µm) (E) and quantitative assessment (F) (*n* = 6). (G) Typical β‐MyHC, ANP, COL‐1 and TGF‐β1 immunoblots in cardiac tissue. GAPDH was the loading reference. (H) The Myh7, Nppa, Cola1 and Tgfb1 transcript expressions in cardiac tissues were measured by RT‐PCR. Actb mRNA was the loading reference (*n* = 6). (I) Typical p‐P65 and IκB‐α western blot assessment in mouse heart cardiac lysates. P65 and GAPDH were the loading references. (J) The Il6 and Tnf transcript expressions in cardiac tissues were analyzed by RT‐qPCR. Actb mRNA was the loading reference (*n* = 6). (K) The serum IL6 and TNF‐α contents as detected by ELISA (*n* = 6). Data provided as mean ± SEM; *p < 0.05; **p < 0.01.

To further validate the critical significance of myeloid CARD9 in ISO‐challenged mice, we carried out the BMT investigation. WT mice were irradiated to deplete BMs and then received transplants of BM from either WT or Card9^−/−^ mice to generate the WT mice with WT BM (WT→WT) and with CARD9–deficient BM (Card9^−/−^→WT). We validated the effectiveness of BM deficiency and reconstitution via examination of Card9 transcript expressions in cardiac tissues and MPMs isolated from these two cohorts (Figure S4). Echocardiography assessment revealed that Card9^−/−^→WT mice were resistant to ISO‐induced changes in systolic dysfunction relative to WT→WT mice (Table 2 and Figure S5A). Transplantation of Card9^−/−^ BM cells to WT mice also inhibited cardiac hypertrophy under ISO infusion compared with the control chimeric mice (Table 2 and Figure 3A–C). The degrees of cardiac structural disorder and fibrosis were strongly decreased in ISO‐infused myeloid Card9‐deficient mice, as indicated by H&E and Sirius Red staining (Figure 3D–F). The β‐MyHC, ANP, COL‐1 and TGF‐β1 protein and mRNA expressions confirmed myeloid CARD9 regulated ISO‐induced cardiac hypertrophy and fibrosis (Figure 3G,H and Figure S5B). CARD9 deletion in myeloid cells also reduced NF‐κB activation and inflammatory gene expression in ISO‐stimulated mouse hearts (Figure 3I–K and Figure S5C,D). These results validated the significance of myeloid CARD9 in ISO‐driven cardiac injuries.

**TABLE 2:** Echocardiographic demographics of experimental mice.

*Model (2)*	WT→WT ISO (*n* = 6)	*Card9* ^−/−^→WT ISO (*n* = 6)
HW/BW (mg/g)	5.59±0.10	4.85±0.12^**^
HW/TL (mg/mm)	7.72±0.14	6.57±0.18^**^
LVIDs (mm)	2.83±0.12	2.50±0.13^ns^
LVIDd (mm)	3.80±0.15	3.64±0.12^ns^
EF%	50.92±1.93	61.76±2.17^**^
FS %	25.53±1.18	32.66±1.54^**^
LV mass (mg)	157.20±9.25	108.0±6.73^**^
LVAWs (mm)	1.58±0.06	1.34±0.06^*^
LVAWd (mm)	1.20±0.07	0.86±0.05^**^

Transthoracic echocardiography was performed on mice at the ending of the animal study.

Abbreviations: BW, body weight; EF, ejection fraction; FS, fractional shortening; HW, heart weight; LVAWd, left ventricular anterior wall end‐diastolic dimension; LVAWs, left ventricular anterior wall end‐systolic dimension; LVIDd, left ventricular end‐diastolic internal diameter; LVIDs, left ventricular end‐systolic internal diameter; ns = not significant.TL, tibial length.

Data presented as Mean & SEM.

* *P* <0.05.

** *P*<0.01 relative to WT→WT ISO.

### 3.4 Macrophage CARD9 blockade prevented ISO‐induced inflammatory responses and then alleviated remodelling changes in cardiomyocytes and fibroblasts

**FIGURE 4:**
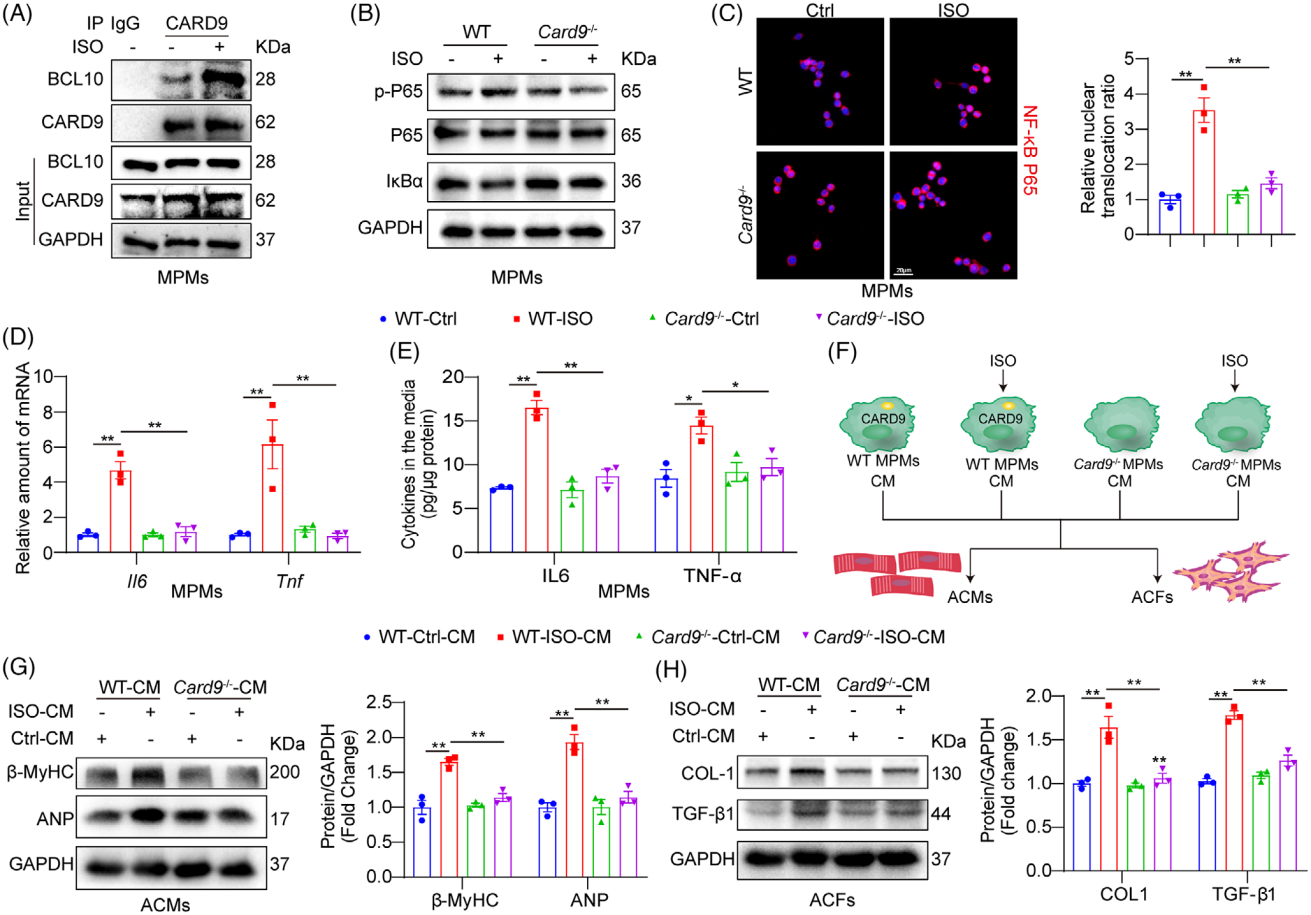
Macrophage CARD9 blockade prevented ISO‐induced inflammatory responses and then alleviated remodelling changes in cardiomyocytes and fibroblasts. (A) MPMs isolated from WT mice underwent 1 h treatment with ISO (20 µM). Following CARD9 IP, BCL10 contents were measured via IB. IgG was the IP control. (B, C) MPMs from Card9^−/−^ and WT mice were induced with ISO (20 µM) over 2 h. Cellular p‐P65 and IκBα were assessed via Western blotting (WB), with P65 and GAPDH as loading references, respectively (B). Immunofluorescence staining targeting the NF‐κB P65 subunit (red) in MPMs. Cells underwent DAPI counterstaining (blue) (scale bar = 20 µm). Quantification values on the right (*n* = 3) (C). (D, E). MPMs from Card9^−/−^ and WT mice were treated with ISO (20 µM) over 24 h. The Il6 and Tnf transcript expressions were detected by RT‐qPCR. Actb mRNA was the loading reference (*n* = 3) (D). The IL6 and TNF‐α cytokines contents in culture media were detected by ELISA (*n* = 3) (E). (F) Depiction of our experimental model examining the significance of ISO‐driven paracrine factors. MPMs from Card9^−/−^ and WT mice were treated with ISO (20 µM), and resulting CM was obtained. ACMs and ACFs were treated with CM over 24 h. (G) β‐MyHC and ANP protein expressions in ACMs lysates, as detected by WB. GAPDH was the control reference (*n* = 3). (H) The COL‐I and TGF‐β1 protein expressions in ACFs whole cell lysates. GAPDH was the control reference (*n* = 3). Data provided as mean ± SEM; *p < 0.05; **p < 0.01.

To validate that macrophage CARD9 mediates ISO‐driven inflammatory response, MPMs derived from WT mice and Card9^−/−^ mice were utilized. ISO stimulation significantly enhanced the interaction between CARD9 and BCL10 in MPMs from WT mice (Figure 4A), indicating CARD9 signalling pathway was activated. ISO stimulation promoted NF‐κB activation in WT MPMs, as evidenced by IκBα degradation, P65 phosphorylation and the nuclear translocation of the P65 subunit, while these changes were blocked in MPMs from Card9^−/−^ mice (Figure 4B,C and Figure S6). CARD9 knockout reduced both the mRNA expression and supernatant levels of inflammatory factors (IL6 and TNF‐α; Figure 4D,E). The intercellular crosstalk between macrophages and cardiac cells was critical in the pathophysiology of cardiac remodelling.^22^ As shown in Figure 4F, we further assessed the influence of macrophage‐derived factors on cardiomyocytes and fibroblasts. We prepared conditioned medium (CM) from WT MPMs or Card9^−/−^ MPMs, exposed to ISO for 24 h. CM from ISO‐exposed WT MPMs increased the levels of hypertrophy‐related proteins in ACMs, as well as fibrosis‐related proteins in ACFs (Figure 4G,H). However, ISO exposure of Card9^−/−^ MPMs was unable to promote pathological alterations in both ACMs and ACFs. Collectively, CARD9 deficiency in macrophages suppressed ISO‐induced inflammation and subsequent intercellular crosstalk, resulting in reduced cardiomyocyte hypertrophy and fibroblast remodelling.

### 3.5 ISO promotes CARD9 activation through OTUD1‐mediated deubiquitinating modification

**FIGURE 5:**
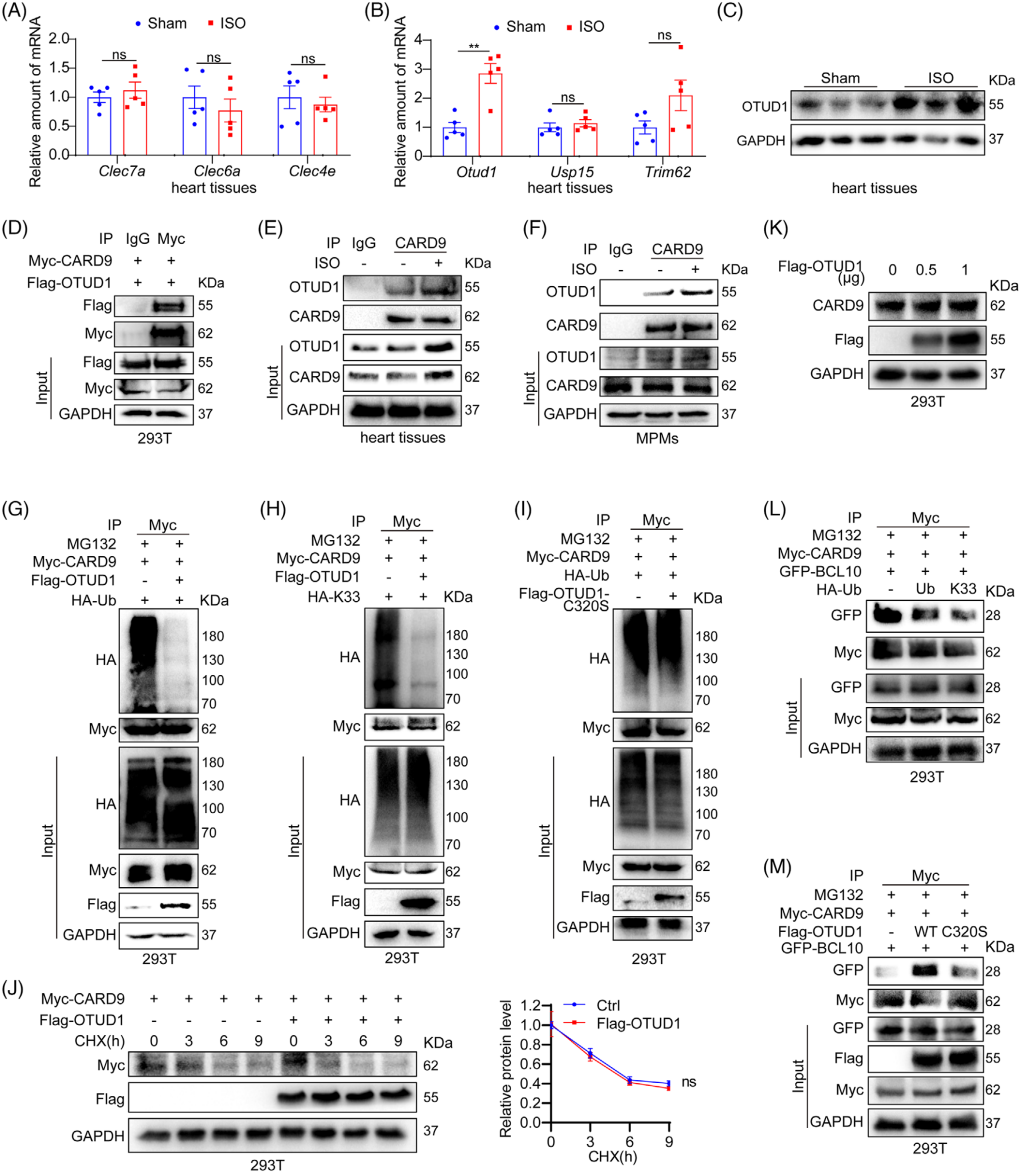
ISO activated CARD9 through OTUD1‐mediated deubiquitinating modification. (A–C) WT mice were provided with 30 mg·kg^−1^·day^−1^ ISO for 2 weeks. The cardiac Clec7a, Clec6a and Clec4e transcript levels were detected by RT‐qPCR. Actb mRNA was the loading reference (*n* = 5) (A). The cardiac Otud1, Usp15 and Trim62 mRNA expressions were detected by RT‐qPCR. Actb mRNA was the loading reference (*n* = 5) (B). Typical cardiac OTUD1 immunoblots. GAPDH was the loading reference (C). (D) HEK‐293T cells were incorporated with Myc‐CARD9 and Flag‐OTUD1. Co‐immunoprecipitation (Co‐IP) assays were performed with anti‐Myc and followed by WB of Flag and Myc. IgG was the IP control. (E) WT mice were provided with 30 mg·kg^−1^·day^−1^ ISO for 2 weeks. ISO‐induced cardiac CARD9 and OTUD1 Co‐IP. Endogenous CARD9 was IP by anti‐CARD9 antibody. IgG was the IP control. (F) MPMs from WT mice underwent a 1 h treatment with ISO (20 µM). Co‐IP assays were performed with anti‐CARD9 and followed by western blot of OTUD1 and CARD9. IgG was the IP control. (G) HEK‐293T cells were incorporated with Myc‐CARD9, Flag‐OTUD1 and HA‐Ub. Cells underwent a 6 h treatment with MG132 (10 µM). Ubiquitinated CARD9 as evidenced by immunoblotting using anti‐HA antibody. (H) HEK‐293T cells were incorporated with Myc‐CARD9, Flag‐OTUD1 and HA‐K33, and then treatment with MG132 (10 µM) for 6 h. Ubiquitinated CARD9 as evidenced by immunoblotting using anti‐HA antibody. (I) HEK‐293T cells were incorporated with Myc‐CARD9, Flag‐OTUD1‐C320S and HA‐Ub, and then treated with MG132 (10 µM) for 6 h. Ubiquitinated CARD9 as evidenced by immunoblotting using anti‐HA antibody. (J) HEK‐293T cells were incorporated with Myc‐CARD9 and Flag‐OTUD1 and then treatment with cycloheximide (25 µg/mL) pulse‐chase stimulation. CARD9 expression using WB analysis. GAPDH was the loading reference (*n* = 3). (K) HEK‐293T were incorporated with varying quantities of Flag‐OTUD1. CARD9 expression using WB analysis. GAPDH was the loading reference. (L) HEK‐293T were incorporated with Myc‐CARD9, GFP‐BCL10, HA‐Ub and HA‐K33, and then treated with MG132 (10 µM) for 6 h. Co‐IP assays were performed with anti‐Myc and followed by a western blot of GFP and Myc. (M) HEK‐293T were incorporated with Myc‐CARD9, GFP‐BCL10, Flag‐OTUD1 and Flag‐OTUD1‐C320S, and then treated with MG132 (10 µM) for 6 h. Co‐IP assays were performed with anti‐Myc and followed by WB of GFP and Myc. Data provided as mean ± SEM; **p < 0.01; ns, not significant.

Next, we aimed to explore how ISO activates CARD9 in macrophages. CARD9 is a critical adapter of C‐type lectin receptors (CLRs), such as Dectin‐1 (Clec7a), Dectin‐2 (Clec6a) and Mincle (Clec4e), in response to fungal, viral, bacterial and parasitic infections.^23^ However, we did not find marked alterations in the transcript expressions of these CLRs within heart tissues infused with ISO (Figure 5A). Post‐translational ubiquitinating modifications directly modulate the CBM complex assembly and activation. The activity of CARD9 has been reported to be regulated by E3 ubiquitin ligase TRIM62 and deubiquitinating enzymes (OTUD1 and USP15) in antifungal immunity.^24^, ^25^, ^26^ Interestingly, we demonstrated that the Otud1 mRNA content was strongly enhanced in ISO‐infused mouse hearts, but not Usp15 and Trim62 (Figure 5B). ISO challenge also markedly enhanced OTUD1 protein levels in mouse hearts (Figure 5C and Figure S7). The interaction of OTUD1 and CARD9 was confirmed in HEK‐293T, heart tissues and MPMs isolated from WT mice, respectively (Figure 5D–F). What's more, ISO stimulation increased OTUD1‐CARD9 complex formation in heart tissues and MPMs (Figure 5E,F). Chen et al.^24^ reported that the K33‐linked polyubiquitin chain was eliminated by OTUD1 from CARD9, which then upregulated the CBM complex. We then determined that OTUD1 could deubiquitinate CARD9 and remove K33‐linked polyubiquitin chains from CARD9 (Figure 5G,H). Cysteine at position 320 in OTUD1 is recognized as a deubiquitinating activity active site.^27^ Indeed, the OTUD1 C320S mutant failed to remove ubiquitin molecules from CARD9 (Figure 5I). We also traced the stability of CARD9 protein in HEK‐293T treated with cycloheximide, a de novo protein synthesis inhibitor. No notable changes were observed in the degradation rate of CARD9 protein in HEK‐293T transfected with Flag‐OTUD1 (Figure 5J). Overexpressing Flag‐OTUD1 in HEK 293T cells did not increase the protein level of CARD9 (Figure 5K). These results showed that OTUD1 has no effect on the CARD9 protein stability. As expected, either total ubiquitination or K33‐linked ubiquitination of CARD9 remarkably hindered the CARD9 and BCL10 association (Figure 5L). OTUD1, but not OTUD1 mutant, enhanced the CARD9‐BCL10 complex formation by deubiquitinating CARD9 (Figure 5M). These results indicate that ISO promotes CARD9 activation through OTUD1‐mediated deubiquitinating modification.

### 3.6 OTUD1 mediates inflammatory responses in macrophages by regulating CARD9–BCL10 interaction

**FIGURE 6:**
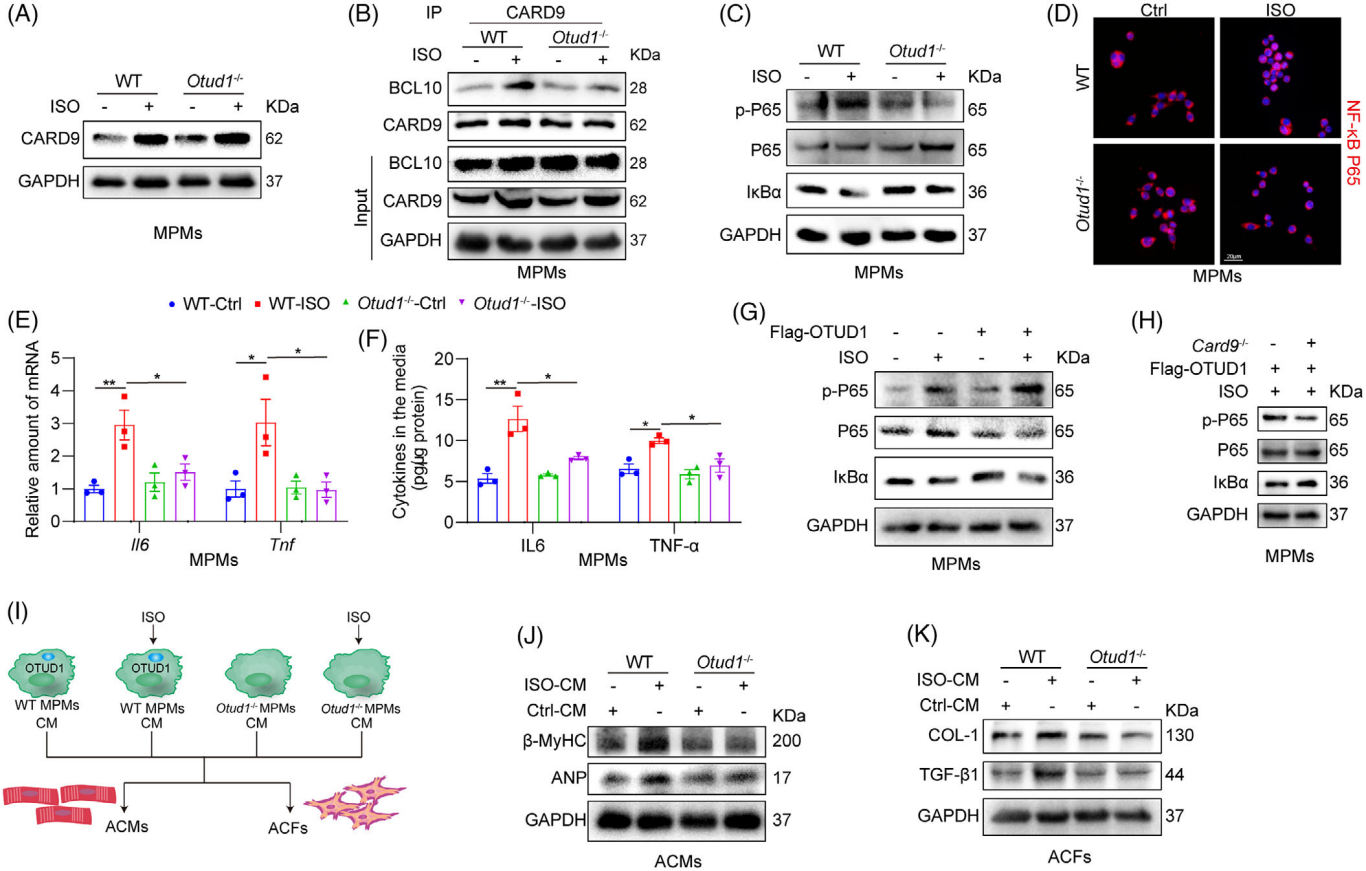
OTUD1 mediates inflammatory responses in macrophages by regulating CARD9‐BCL10 interaction. (A) MPMs from Otud1^−/−^ mice and WT mice underwent a 12 h treatment with ISO (20 µM). Typical cellular CARD9 immunoblots. GAPDH was the loading reference. (B) MPMs from Otud1^−/−^ and WT mice underwent a 1 h treatment with ISO (20 µM). Typical immunoblots depicting CARD9 and BCL10 co‐immunoprecipitation. Endogenous CARD9 was IP by the anti‐CARD9 antibody. (C, D) MPMs from Otud1^−/−^ and WT mice underwent a 2 h treatment with ISO (20 µM). Cellular p‐P65 and IκBα were assessed via WB, with P65 and GAPDH as loading references, respectively (C). Immunofluorescence staining targeting the NF‐κB P65 subunit (red) in macrophages. Cells underwent DAPI counterstaining (blue) (scale bar = 20 µm) (D). (E,F) MPMs from Otud1^−/−^ and WT mice underwent a 24 h treatment with ISO (20 µM). The Il6 and Tnf mRNA expressions as detected by RT‐PCR. Actb mRNA was the loading reference (*n* = 3) (E). The IL6 and TNF‐α cytokines contents in culture media as detected by ELISA (*n* = 3) (F). (G) MPMs from WT mice were incorporated with Flag‐OTUD1, prior to a 2 h treatment with ISO (20 µM). Cellular p‐P65 and IκBα as detected by WB, with P65 and GAPDH as loading references, respectively. (H) MPMs from Card9^−/−^ and WT mice were incorporated with Flag‐OTUD1, prior to a 2 h treatment with ISO (20 µM). Cellular p‐P65 and IκBα as detected by WB, with P65 and GAPDH as loading references, respectively. (I) Depiction of our experimental model examining the significance of ISO‐driven paracrine factors. MPMs from Otud1^−/−^ and WT mice underwent a 24 h treatment with ISO (20 µM). CM was obtained. The ACMs and ACFs underwent a 24 h treatment with CM. (J) β‐MyHC and ANP contents in ACMs lysates as detected by WB. GAPDH was the reference (*n* = 3). (K) COL‐I and TGF‐β1 expressions in whole ACF lysates. GAPDH was the reference (*n* = 3). Data provided as mean ± SEM; *p < 0.05; **p < 0.01.

We then determined whether OTUD1 could mediate ISO‐induced inflammatory responses in macrophages by regulating CARD9. We observed that OTUD1 knockout did not affect CARD9 protein level, but significantly reduced ISO‐induced CARD9–BCL10 interaction in ISO‐challenged macrophages (Figure 6A,B). OTUD1 deficiency further inhibited the activation of NF‐κB, reduced Il6 and Tnf transcript levels and limited the protein release of IL6 and TNF‐α in ISO‐stimulated MPMs (Figure 6C–F and Figure S8). Contrarily, OTUD1 overexpression exacerbated ISO‐driven NF‐κB activation (Figure 6G and Figure S9). Interestingly, NF‐κB activation induced by OTUD1 overexpression in MPMs was significantly limited in Card9 knockout mice, indicating OTUD1 regulated NF‐κB activity through CARD9 (Figure 6H and Figure S10). We also treated MPMs from WT or Otud1^−/−^ mice with ISO and obtained the CM of MPMs to incubate cardiomyocytes or fibroblasts (Figure 6I). As seen in Figure 6J,K and Figure S11, the CM from ISO‐stimulated Otud1^−/−^ MPMs could not promote hypertrophy in ACMs and fibrosis in ACFs.

### 3.7 OTUD1 deletion in myeloid cells protected hearts from ISO‐induced injuries in mice

**FIGURE 7:**
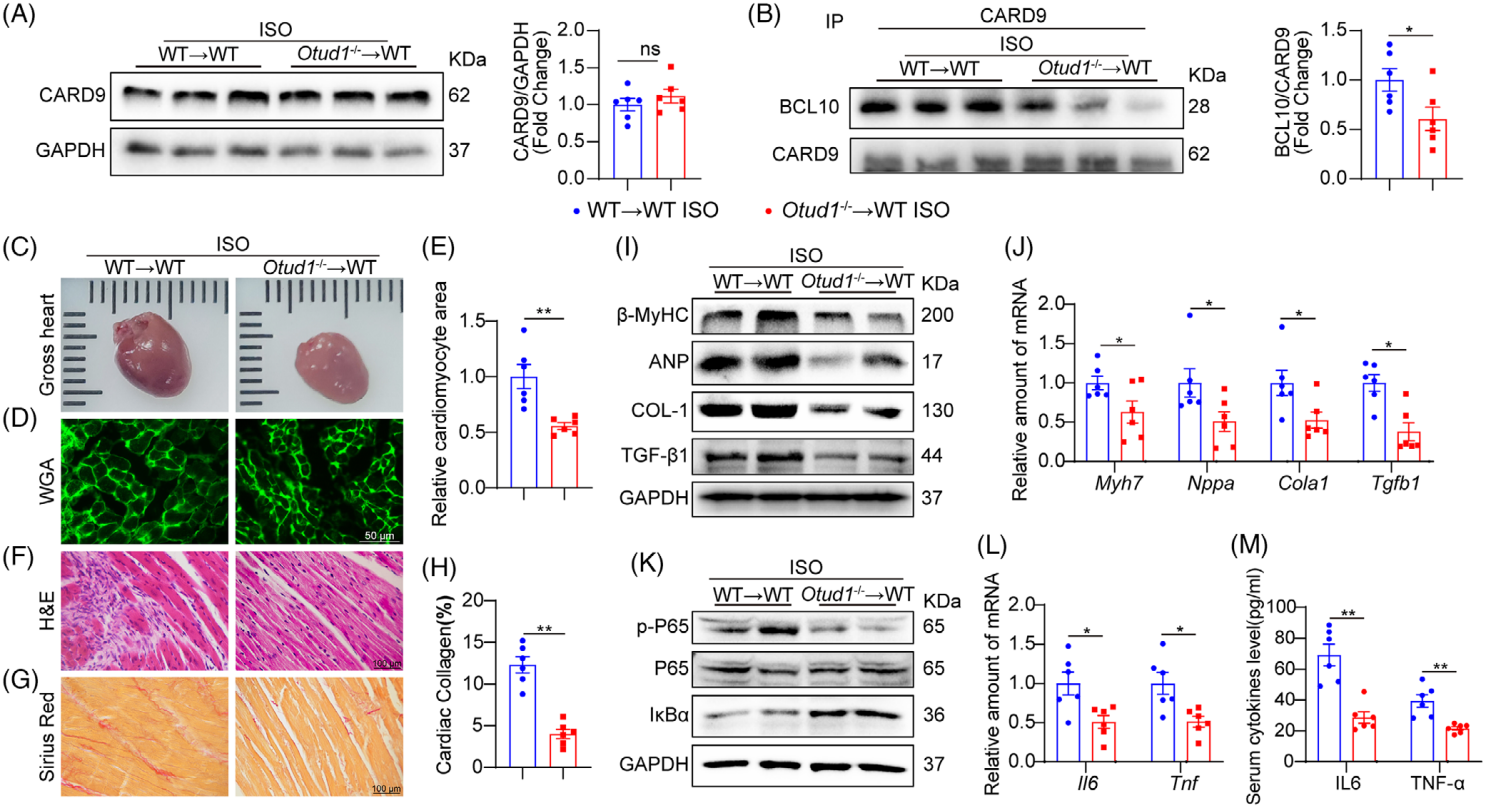
OTUD1 deletion in myeloid cells protected hearts against ISO‐induced injuries in mice. WT mice underwent irradiation and received BMs from either WT or Otud1^−/−^ mice. Mice were provided with 30 mg·kg^−1^·day^−1^ ISO over 2 weeks. (A) Typical cardiac CARD9 immunoblots. GAPDH was the loading reference. Quantification provided on the right (*n* = 6). (B) Typical immunoblots depicting cardiac CARD9 and BCL10 co‐IP. Endogenous CARD9 was IP using an anti‐CARD9 antibody. IgG was the IP control. Quantification provided on the right (*n* = 6). (C) Typical gross heart images. (D,E) Cardiomyocyte areas were evaluated via WGA staining (scale bar = 50 µm) (D) and quantitative assessment (E) of cardiac sections (*n* = 6). (F) Typical cardiac tissues H&E staining (scale bar = 100 µm). (G,H) Typical cardiac sections Sirius Red staining (scale bar = 100 µm) (G) and quantitative assessment (H) (*n* = 6). (I) Typical cardiac β‐MyHC, ANP, COL‐1 and TGF‐β1 immunoblots. GAPDH was the loading reference. (J) The cardiac Myh7, Nppa, Cola1 and Tgfb1 mRNA expressions as detected by RT‐PCR. Actb mRNA was the loading reference (*n* = 6). (K) Typical p‐P65 and IκBα western blot analysis in mouse cardiac tissue lysates. P65 and GAPDH were the loading references. (L) The cardiac Il6 and Tnf mRNA expression were detected by RT‐PCR. Actb mRNA was the loading reference (*n* = 6). (M) Serum IL6 and TNF‐α contents as detected by ELISA (*n* = 6). Data provided as mean ± SEM; *p < 0.05; **p < 0.01.

To elucidate the macrophage OTUD1 contribution in regulating ISO‐driven cardiac injuries, we administered BM from either WT mice (WT→WT mice) or Otud1^−/−^ mice (Otud1^−/−^→WT mice) to WT mice. These mice were implanted with osmotic pumps continuously releasing ISO to induce cardiac inflammation and remodelling. Isolated MPMs and heart tissues were used to confirm the specific deletion of OTUD1 (Figure S12). As expected, myeloid OTUD1 deficiency did not affect CARD9 stability but suppressed CARD9‐BCL10 interaction in heart tissues under ISO stimulation (Figure 7A,B). Noninvasive echocardiography showed that OTUD1 deficiency significantly inhibited ISO‐induced cardiac dysfunction (Table 3 and Figure S13A). Echocardiography results, along with measurements of HW/BW, HW/TL, gross heart size, cardiomyocyte size and interstitial fibrosis, indicated that myeloid‐specific OTUD1 knockout abrogated ISO‐driven cardiac hypertrophy and fibrosis (Table 3 and Figure 7C–H). These results were further validated by assessing the β‐MyHC, ANP, COL‐1 and TGF‐β1 protein and mRNA expressions (Figure 7I–J and Figure S13B). Myeloid‐specific OTUD1 knockout also inhibited NF‐κB activation and inflammatory cytokine overproduction in ISO‐challenged mice (Figure 7K–M and Figure S13C,D). These findings provided evidence that myeloid OTUD1 mediated ISO‐induced CARD9‐BCL10 interaction, cardiac inflammation, hypertrophy and dysfunction in mice.

**TABLE 3:** Echocardiographic demographics of experimental mice.

*Model (3)*	WT→WT ISO (*n* = 6)	*Otud1* ^−/−^→WT ISO (*n* = 6)
HW/BW(mg/g)	5.83±0.18	5.20±0.19^*^
HW/TL(mg/mm)	7.23±0.19	5.48±0.14^**^
LVIDs(mm)	2.92±0.15	2.36±0.16^*^
LVIDd(mm)	3.94±0.15	3.51±0.18^ns^
EF%	51.59±2.65	61.92±2.51^*^
FS %	26.06±1.61	32.71±1.78^*^
LV mass (mg)	176.00±18.07	103.7±10.22^**^
LVAWs (mm)	1.63±0.07	1.38±0.08^*^
LVAWd (mm)	1.15±0.07	0.92±0.04^*^

Transthoracic echocardiography was performed on mice at the ending of the animal study.

Abbreviations: BW, body weight; EF, ejection fraction; FS, fractional shortening; HW, heart weight; LVAWd, left ventricular anterior wall end‐diastolic dimension; LVAWs, left ventricular anterior wall end‐systolic dimension; LVIDd, left ventricular end‐diastolic internal diameter; LVIDs, left ventricular end‐systolic internal diameter; ns = not significant; TL, tibial length.

Data presented as Mean & SEM.

* *P* <0.05.

** *P*<0.01 relative to WT→WT ISO.

## 4 DISCUSSION

Recent studies have demonstrated that CARD9 is crucial in the development and progression of various cardiovascular diseases, including TAC‐induced HF, myocardial ischemia/reperfusion, hypertension and atherosclerosis.^15^, ^28^, ^29^, ^30^ For example, Wang et al.^31^ showed that the contributions of CARD9 and BCL10 in obesity‐related cardiac hypertrophy. The chronic ISO model presents symptom characteristics of advanced HF, with chronically elevated levels of catecholamines mimicking chronic adrenergic stimulation. Herein, we examined the CARD9 significance in ISO‐driven pathological cardiac remodelling and revealed the associated signalling network. We detected an increased expression of macrophage CARD9 in heart tissues following ISO infusion, suggesting that CARD9 is involved in HF. Furthermore, either whole‐body CARD9 knockout or myeloid‐specific CARD9 deletion attenuated ISO‐driven cardiac dysfunction, inflammation, hypertrophy and fibrosis. We found that ISO promoted the assembly of CBM complex to trigger NF‐κB activation and proinflammatory responses. Importantly, we found that ISO‐activated CARD9 via a deubiquitinase OTUD1, which has been implicated in pathological cardiac remodelling.^32^ We demonstrate that OTUD1 binds to the CARD9 and removes the K33‐linked ubiquitin from CARD9, thus promoting the assembly of the CBM complex in macrophages upon ISO stimulation. The macrophage inflammation mediated by the ISO‐OTUD1‐CARD9 axis further promotes hypertrophy and fibrosis in cardiomyocytes and fibroblasts, respectively, inducing cardiac remodelling. Taken together, our findings suggested that targeting the OTUD1‐CARD9 axis has a protective effect against ISO‐driven cardiac inflammation and HF.

Inflammation participates in the progression of ISO‐induced HF. It was well established that β‐AR blockers exposure reduced inflammation in chronic HF patients.^6^ Elucidating the signalling pathways involved in ISO‐induced inflammatory responses is of great importance. Some CARD family members have been shown to mediate inflammatory cardiomyopathy.^33^, ^34^ Card9 mRNA levels were the most significantly elevated among all Card family genes in the hearts of mice under ISO infusion. CARD9‐based network strongly regulates inflammatory responses via upregulation of cytokines and chemokines synthesis. Inflammation further promotes macrophage infiltration, activation and cytokines release, enhancing oxidative stress and subsequent tissue injuries within hearts using a positive feedback loop.^35^ Here, we found that macrophage CARD9 deficiency inhibited ISO‐driven NF‐κB activation and inflammatory responses and subsequent cardiomyocyte hypertrophy and fibroblast fibrosis.

Both protein phosphorylation and ubiquitination regulate CARD9 activation. PKC‐δ directly phosphorylates threonine at position 231 of CARD9, and this modification is necessary for the CBM complex assembly.^36^ E3 ubiquitin ligase TRIM62 associates with the CARD9 C‐terminal, ubiquitinating CARD9 in a K23‐linked manner and then promoting the interaction of CARD9‐BCL10 in anti‐fungal immunity and intestinal inflammation.^26^ Here, we found that OTUD1 deubiquitinated CARD9 in ISO‐challenged macrophages and mouse hearts. OTUD1 eliminated K29‐, K33‐ and K63‐linked polyubiquitination from CARD9, but only the removal of K33‐linked CARD9 polyubiquitination promoted CBM complex assembly in antifungal immunity.^24^ Our results validated that OTUD1 has no effect on the CARD9 stability. OTUD1 promotes CBM complex formation and CBM‐mediated inflammation by catalyzing the K33‐linked deubiquitination of CARD9 in ISO‐challenged macrophages, which further contributes to inflammatory heart remodelling.

OTUD1, a deubiquitinase from the OTU protein family, is essential in regulating antiviral host defence, tumour progression and inflammation.^37^, ^38^, ^39^ Recent evidence suggested the OTUD1 significance in cardiovascular diseases. Lu et al.^40^ found that OTUD1 may regulate B lymphocytes and dendritic cells, contributing to doxorubicin‐induced cardiomyopathy. Our group has recently found that cardiomyocyte OTUD1 promoted angiotensin II‐ and TAC‐driven cardiac dysfunction, hypertrophy and fibrosis, while the role of OTUD1 was evaluated using the whole‐body OTUD1 knockout mice but not the myocardial‐specific OTUD1 knockout mice or BMT.^32^ That is to say, the involvement of macrophage OTUD1 in cardiac remodelling has not yet been explored. This research for the first time identified macrophage OTUD1 as an indispensable mediator in ISO‐driven cardiac inflammation, hypertrophy and fibrosis. Macrophage OTUD1 mediates ISO‐driven inflammatory responses by deubiquitinating CARD9 and increasing the combination of CARD9 and BCL10.

The cellular mechanism linking CARD9 to CLRs, specifically through the Dectin‐1 signalling pathway, has been well‐documented in the context of fungal and parasitic infections.^41^, ^42^ CLRs have also been reported to be implicated in sterile inflammation.^43^, ^44^ The CLRs’ significance in ISO‐driven cardiac injury is undetermined. We found that the mRNA levels of CLRs did not change, indicating that CLRs may not affect ISO‐induced cardiac remodelling. In addition to CLR signalling, CARD9 has also been associated with toll‐like receptor (TLR)‐related signalling.^45^ Dendritic cell lacking CARD9 produced reduced levels of cytokines when stimulated by TLR4 or TLR2 stimulation. Rhoads et al.^46^ found that oxidized low‐density lipoprotein immune complexes could induce CBM complex via TLR4 in chronic inflammatory diseases, such as systemic lupus erythematosus, rheumatoid arthritis, atherosclerosis and type‐2 diabetes. TLR2 and TLR4 have been reported to mediate ISO‐induced cardiac inflammation.^18^, ^47^ Therefore, further research is required to determine whether TLR2/4 play a role in regulating CARD9 in ISO‐challenged heart tissues.

This study has some limitations. CARD9 is expressed not only in macrophages but also in neutrophils and dendritic cells. However, our BMT experiments cannot rule out the role of CARD9 on these myeloid cells. Meanwhile, CARD9 expression has also been demonstrated to increase in H9C2 cells and neonatal rat primary cardiomyocytes exposed to hypoxia and hypoxia/reoxygenation, affording CARD9's protection of myocardium against ischemia/reperfusion injury.^29^, ^48^ Thus, the potential role of cardiomyocyte CARD9 in ISO‐induced HF warrants further investigation. A technical limitation of this study is that our experimental conditions limited us from conducting invasive LV hemodynamic testing, which can provide precise data on the heart's pumping efficiency and overall function. It has been observed that both dP/dtmax and dP/dtmin significantly decrease under ISO infusion, indicating that ISO impairs both the systolic and diastolic functions of the heart, leading to HF.^49^, ^50^ Finally, the role of gender differences in cardiac injury under ISO stimulation has attracted widespread attention. Angela et al.^51^ have found that ISO infusion‐treated male rats had more cardiac hypertrophy, fibrosis and mortality than female rats. Here, our research only used male mice and the applicability of the study may be sex‐limited.

## 5 CONCLUSION

In summary, we have demonstrated that the macrophage OTUD1‐CARD9 axis mediates ISO‐induced inflammatory response, subsequently contributing to heart remodelling. We identified that OTUD1 facilitates CBM complex formation through K33‐linked deubiquitination on CARD9. These evidence highlighted the OTUD1‐CARD9 axis in ISO‐driven HF and suggested that targeting OTUD1 or CARD9 could have therapeutic potential for this condition.

## AUTHOR CONTRIBUTIONS

Guang Liang conceived and designed the study. Guang Liang, Gaojun Wu, and Yi Wang supervised this study. Jinfu Qian, Qinyan Wang, Jiachen Xu, Shiqi Liang, Qingsong Zheng, Xiaocheng Guo, and Wu Luo performed the experiments. Weijian Huang, Xiaohong Long, and Julian Min provided professional guidance and technical support. Jinfu Qian, Qinyan Wang, and Jiachen Xu analyzed the data. Jinfu Qian and Guang Liang wrote the manuscript. Guang Liang, Gaojun Wu, Yi Wang, and Jinfu Qian edited and reviewed the manuscript.

## CONFLICT OF INTEREST STATEMENT

The authors declare no conflict of interest.

## ETHICS STATEMENT

All mice protocols received approval by the Laboratory Animal Ethics Committee of Wenzhou Medical University (Approval No. wydw2021‐0438) and abided the guidelines established by the National Institutes of Health.

## Supporting information

Supporting Information

## Data Availability

All relevant data are included in the article or provided as Supporting Information. Additional data are available upon request from the corresponding author.
