# Supplementary material for: Macrophage OTUD1‐CARD9 axis drives isoproterenol‐induced inflammatory heart remodelling
Source: Clin Transl Med. 2024 Aug 8;14(8):e1790. doi: 10.1002/ctm2.1790 (PMC11310286; doi:10.1002/ctm2.1790)
Supplement: Supplementary file 1 — Supporting Information [file CTM2-14-e1790-s001.docx]

***Supplementary Materials***

**Macrophage OTUD1-CARD9 axis drives Isoproterenol-induced inflammatory heart failure**

**List of Supplementary Materials**

Expanded Methods

Figure S1 to S13

Table S1 to S2


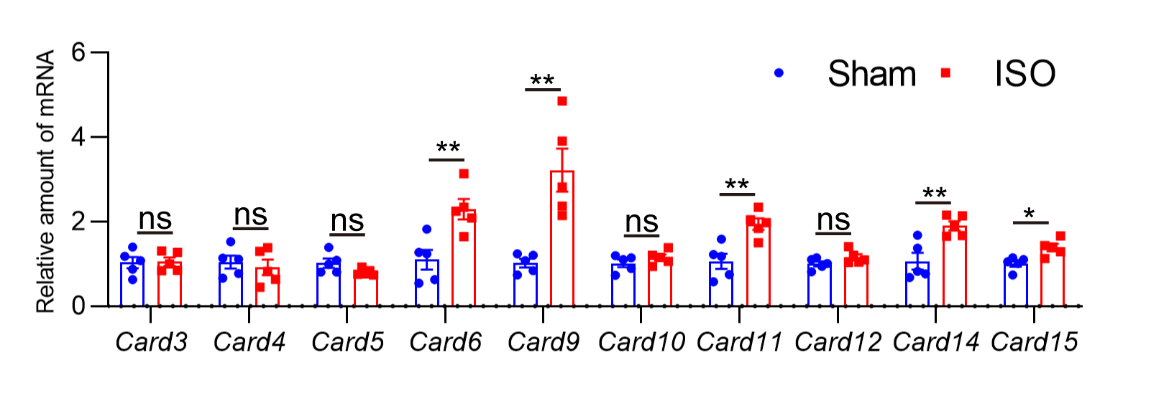


**Supplementary Figure S1: The mRNA levels of *Card9* were increased in mouse hearts tissues following ISO infusion.**

WT mice were administered 30 mg·kg^−1^·d^−1^ ISO for two weeks. The mRNA levels of *Card* family genes in cardiac tissues. *Actb* mRNA was used as loading control (*n* = 5). Data were shown as mean ± SEM; **P* < 0.05; ***P* < 0.01; ns = not significant.


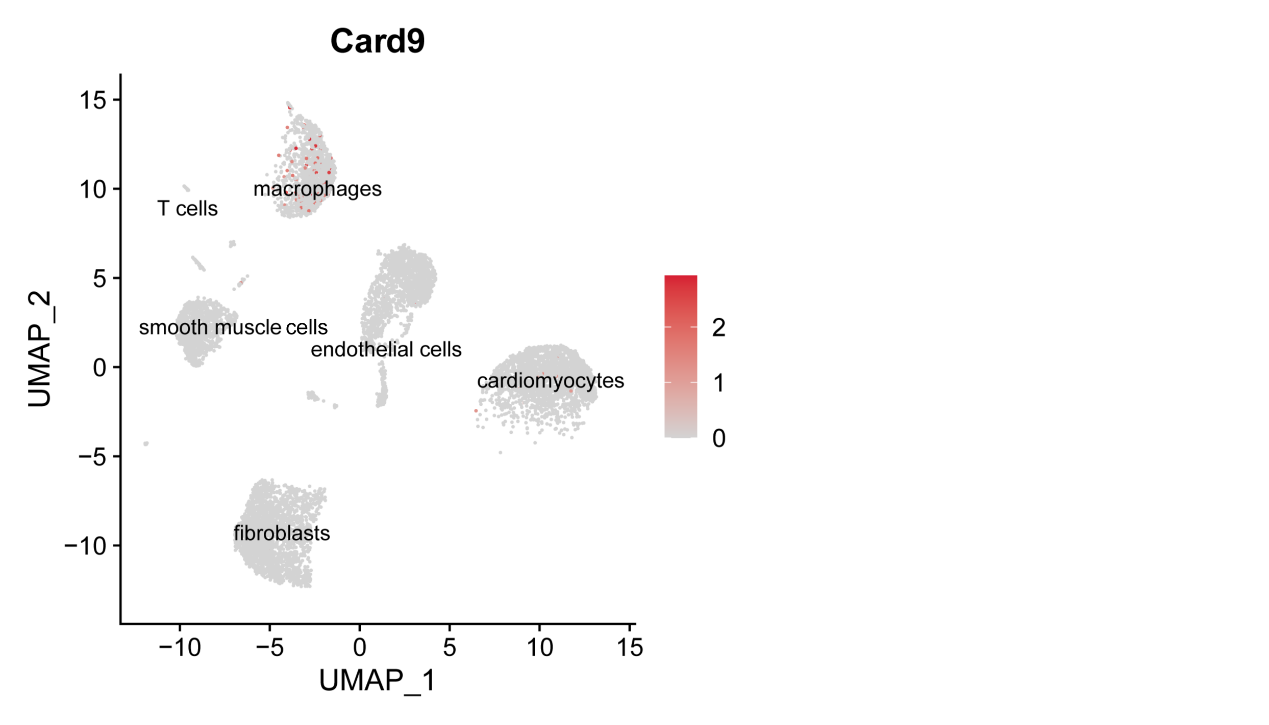


**Supplementary Figure S2: The expression of *Card9* mRNA was predominantly localized within macrophages in the cardiac tissue of Sham group.**

The UMAP plot showed that the *Card9* mRNA expression in 6 main cell types, including macrophages, cardiomyocytes, fibroblasts, endothelial cells, smooth muscle cells, and T cells.


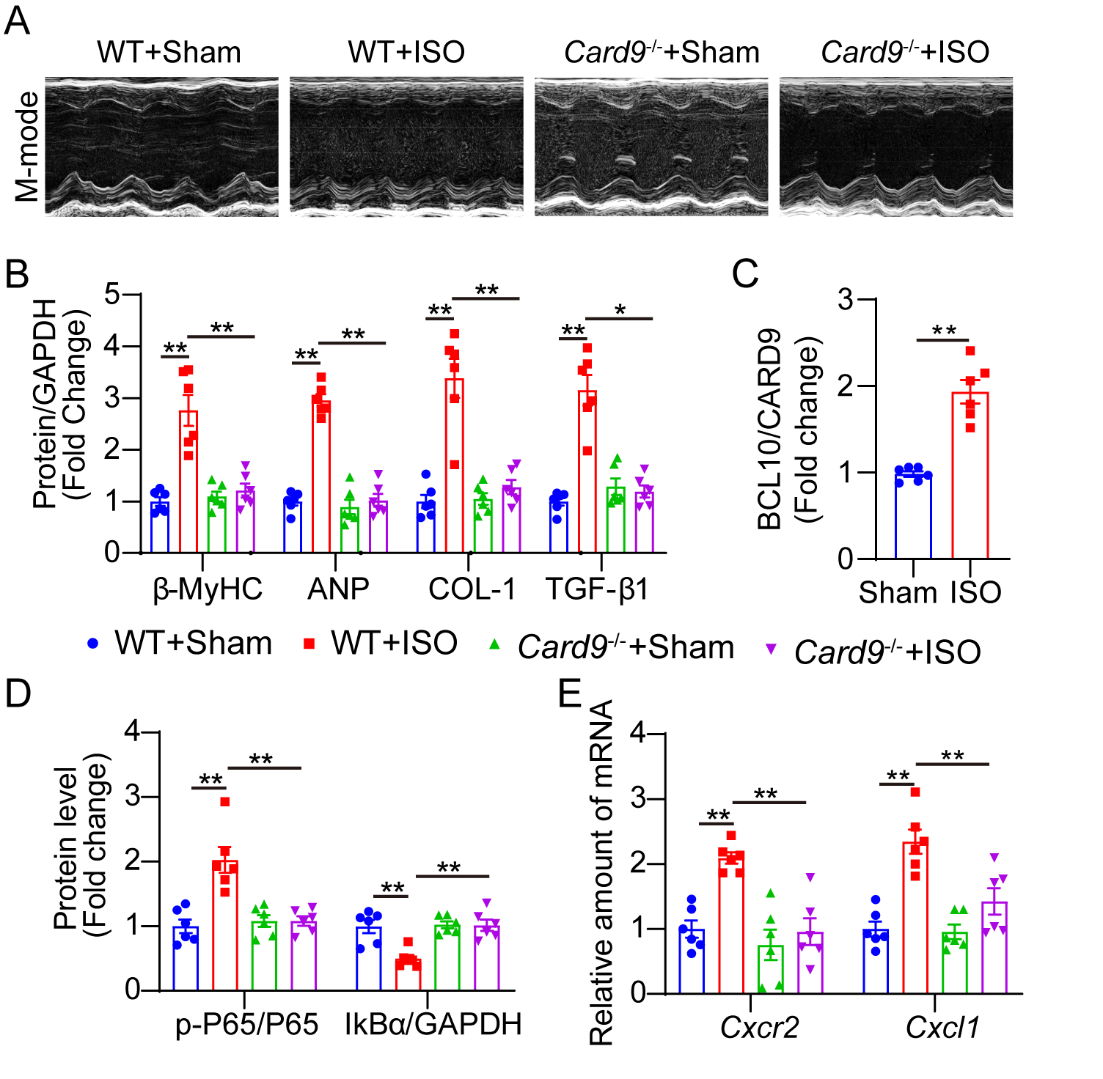


**Supplementary Figure S3: CARD9 deficiency attenuated ISO-induced cardiac injury.**

*Card9^-/-^* mice and WT mice were administered 30 mg·kg^−1^·d^−1^ ISO or an equal volume of sterile water for two weeks. (A) Representative M-mode echocardiographic images of left ventricle. (B) Densitometric quantification of immunoblots in Fig. 2G (*n* = 6). (C) Densitometric quantification of immunoblots in Fig. 2I (*n* = 6). (D) Densitometric quantification of immunoblots in Fig. 2J (*n* = 6). (E) The mRNA levels of *Cxcr2* and *Cxcl1* in heart tissues were determined using RT-qPCR. *Actb* mRNA was used as a loading control (*n* = 6). Data were shown as mean ± SEM; **P* < 0.05; ***P* < 0.01.


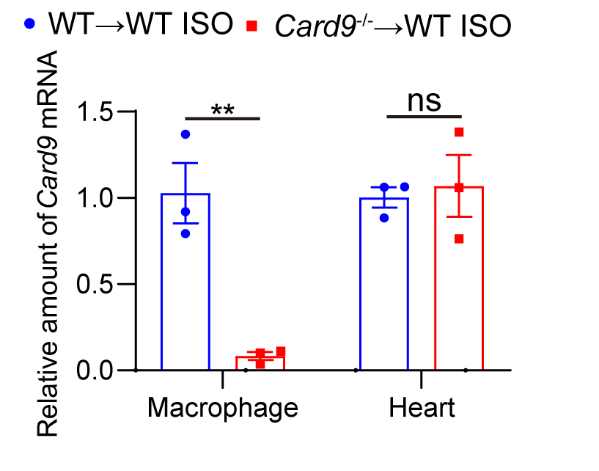


**Supplementary Figure S4: Bone marrow transplant has been established successfully.**

The mRNA levels of *Card9* in isolated macrophages and heart tissues were determined using RT-qPCR. *Actb* mRNA was used as a loading control (*n* = 3). Data were shown as mean ± SEM; ***P* < 0.01; ns=no significant.


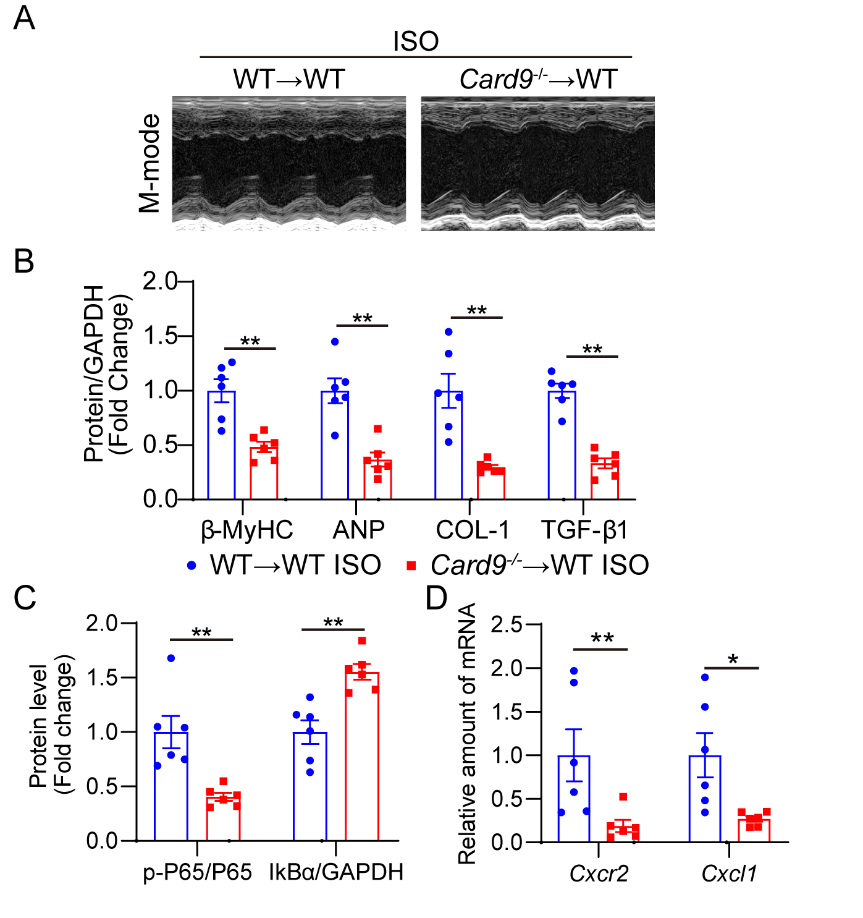


**Supplementary Figure S5: Marrow-derived cell CARD9 mediated ISO-induced cardiac injuries.**

WT mice were irradiated and administered bone marrow cells from either WT or *Card9^-/-^* mice. Mice were then administered 30 mg·kg^−1^·d^−1^ ISO for 2 weeks. (A) Representative M-mode echocardiographic images of left ventricle. (B) Densitometric quantification of immunoblots in Fig. 3G (*n* =6). (C) Densitometric quantification of immunoblots in Fig. 3I (*n* =6). (D) The mRNA levels of *Cxcr2* and *Cxcl1* in heart tissues were determined using RT-qPCR. *Actb* mRNA was used as a loading control (*n* = 6). Data were shown as mean ± SEM; **P* < 0.05; ***P* < 0.01.


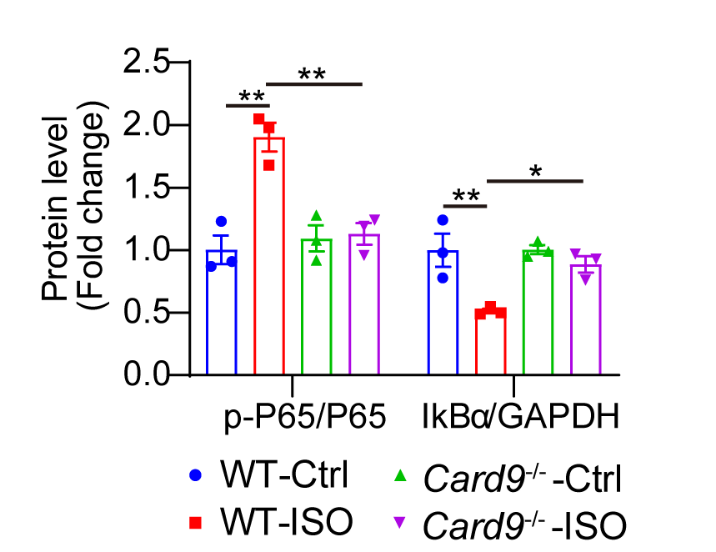


**Supplementary Figure S6: Macrophage CARD9 blockade prevented ISO-induced inflammatory responses in vitro.**

Densitometric quantification of immunoblots in Fig. 4B (*n* =3). Data were shown as mean ± SEM; **P* < 0.05; ***P* < 0.01.


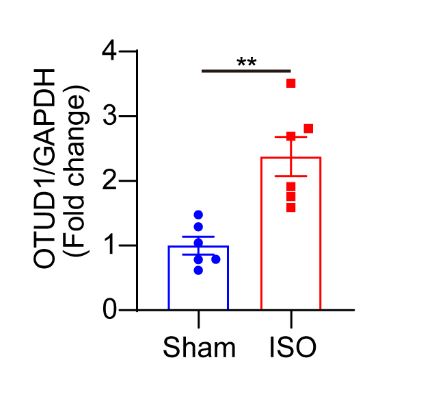


**Supplementary Figure S7: The expression of OTUD1 was up-regulated in heart tissues upon ISO infusion.**

Densitometric quantification of immunoblots in Fig. 5C (*n* =6). Data were shown as mean ± SEM; ***P* < 0.01.


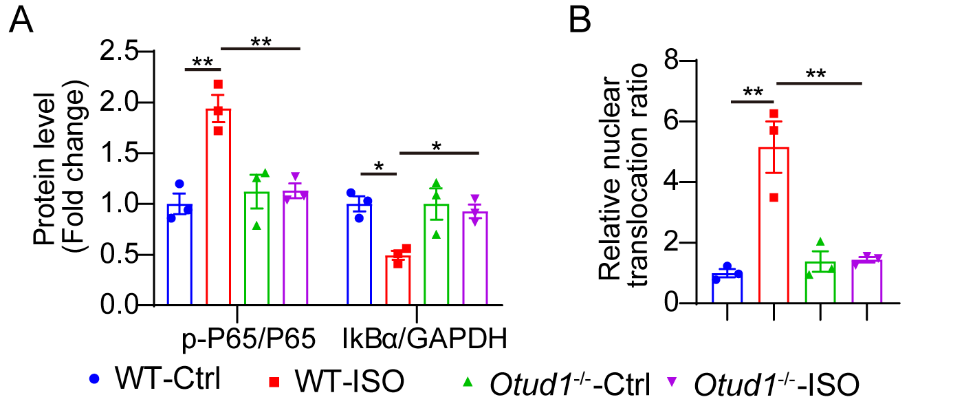


**Supplementary Figure S8: OTUD1 deficiency inhibited ISO–induced inflammatory responses in macrophages.**

(A) Densitometric quantification of immunoblots in Fig. 6C (*n* =3). (B) Quantification of intensity in Fig. 6D (*n* =3). Data were shown as mean ± SEM; **P* < 0.05; ***P* < 0.01.


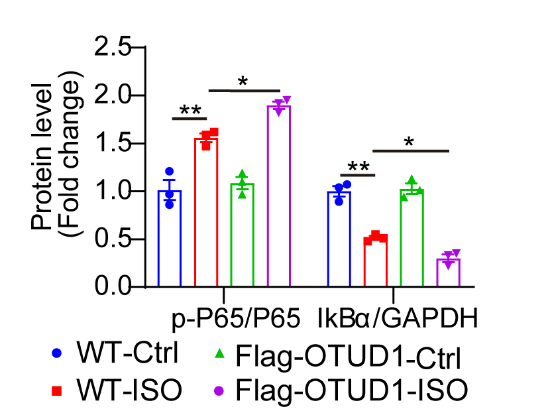


**Supplementary Figure S9: OTUD1 overexpression accelerated ISO–induced inflammatory responses in macrophages.**

Densitometric quantification of immunoblots in Fig. 6G (*n* =3). Data were shown as mean ± SEM; *n* = 3; * *P* < 0.05, ** *P* < 0.01.


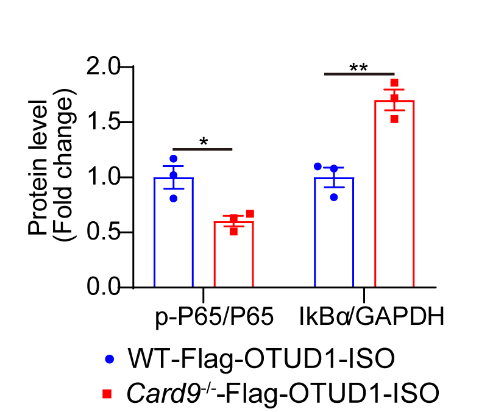


**Supplementary Figure S10: NF-κB activation induced by OTUD1 overexpression in MPMs was significantly limited in *Card9* knockout mice.**

Densitometric quantification of immunoblots in Fig. 6H (*n* =3). Data were shown as mean ± SEM; * *P* < 0.05, ** *P* < 0.01.


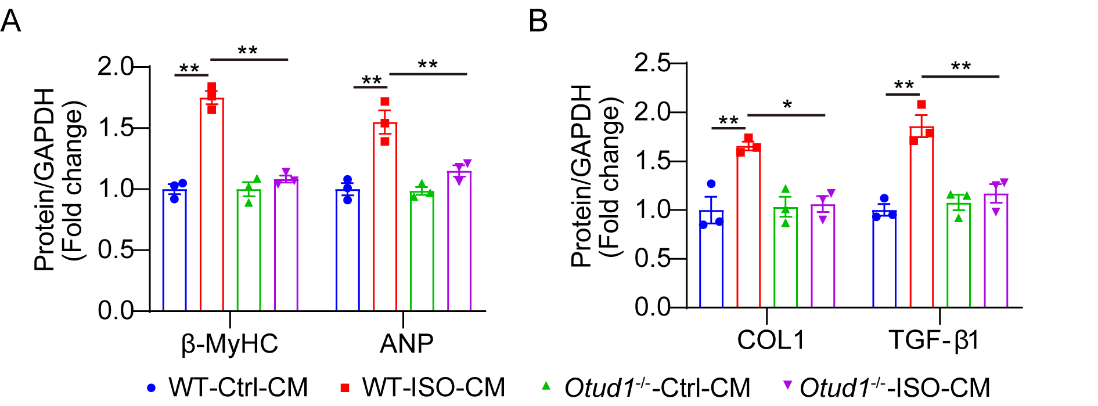


**Supplementary Figure S11: Macrophage OTUD1 deficiency suppressed the cellular crosstalk between macrophages and other cardiac cells.**

(A) Densitometric quantification of immunoblots in Fig. 6J (*n* =3). (B) Densitometric quantification of immunoblots in Fig. 6K (*n* =3). Data were shown as mean ± SEM; **P* < 0.05; ***P* < 0.01.


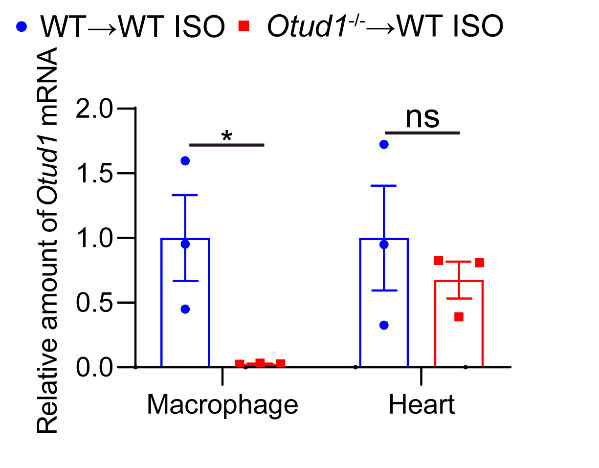


**Supplementary Figure S12: Bone marrow transplant has been established successfully.**

The mRNA levels of *Otud1* in isolated macrophages and heart tissues were determined using RT-qPCR. *Actb* mRNA was used as a loading control (*n* =3). Data were shown as mean ± SEM; **P* < 0.05; ns=no significant.


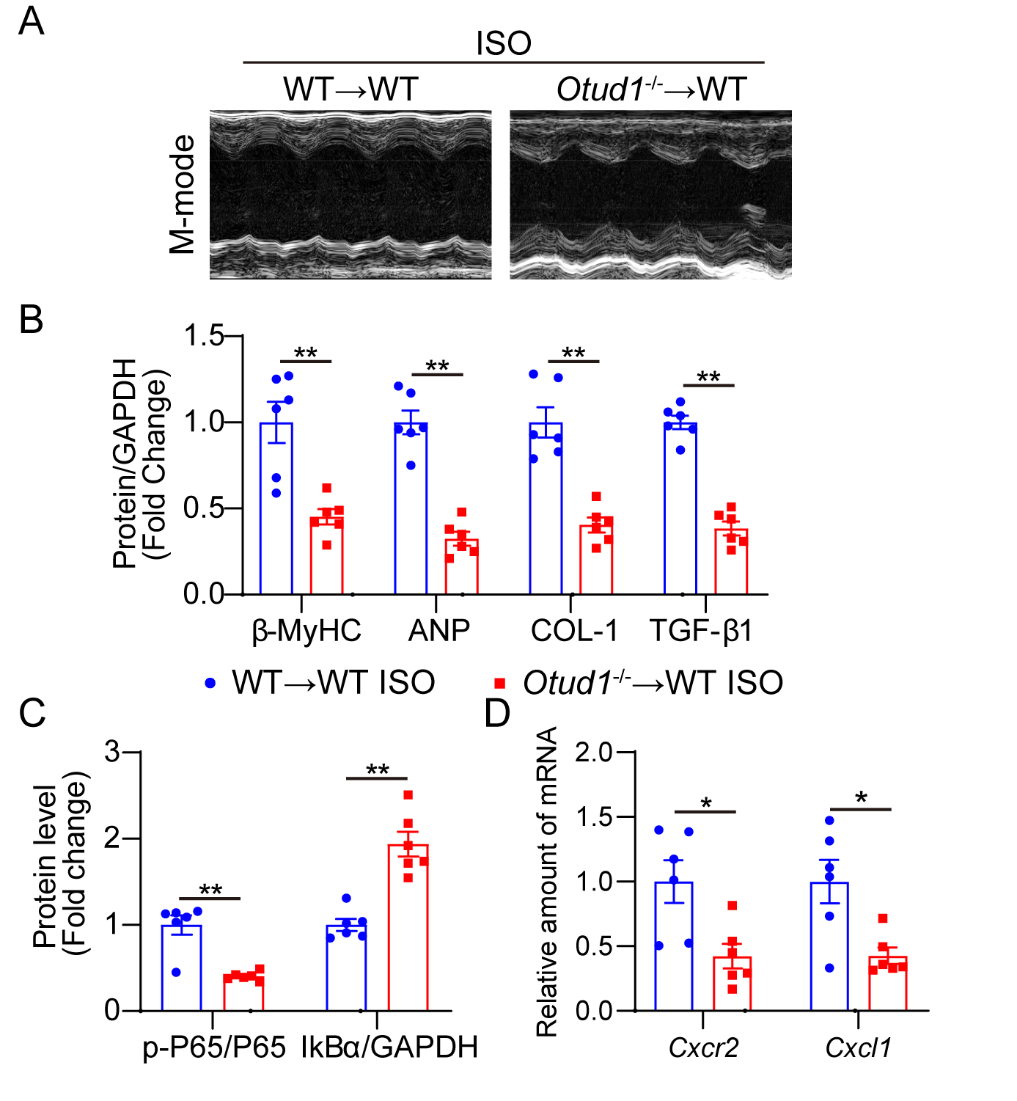


**Supplementary Figure S13: Bone marrow-derived macrophage OTUD1 deficiency decreased ISO-induced cardiac remodeling.**

WT mice were irradiated and administered bone marrow cells from either WT or *Otud1^-/-^* mice. Mice were then administered 30 mg·kg^−1^·d^−1^ ISO for 2 weeks. (A) Representative M-mode echocardiographic images of left ventricle. (B) Densitometric quantification of immunoblots in Fig. 7I (*n* =6). (C) Densitometric quantification of immunoblots in Fig. 7K (*n* =6). (D) The mRNA levels of *Cxcr2* and *Cxcl1* in heart tissues were determined by RT-qPCR. *Actb* mRNA was used as a loading control (*n* =6). Data were shown as mean ± SEM; **P* < 0.05; ***P* < 0.01.

**Supplementary Table S1. Reagent list used in this study.**

| Reagent | Source | Catalogue |
| --- | --- | --- |
| DAPI | Beyotime Biotech | C1006 |
| Lipofectamine 3000 | Thermo Fisher | L3000015 |
| Isoprenaline | Med Chem Express | HY-B0468 |
| MG132 | Med Chem Express | HY-13259 |
| CHX | Med Chem Express | HY-12320 |
| GAPDH antibody | Santa Cruz Biotechnology | sc-365062 |
| CARD9 antibody | Santa Cruz Biotechnology | sc-374569 |
| Goat anti-mouse IgG H&L (TRITC) antibody | Abcam | ab6786 |
| Goat anti-rabbit IgG H&L (Alexa Fluor 488) antibody | Abcam | ab150077 |
| F4/80 antibody | Cell Signaling Technology | 30325 |
| phospho-NFκB P65 (Ser536) antibody | Cell Signaling Technology | 3033 |
| NF-κB P65 antibody | Cell Signaling Technology | 8242 |
| BCL10 | Cell Signaling Technology | 4237 |
| HRP-conjugated goat anti-mouse IgG antibody | Cell Signaling Technology | 7076 |
| HRP-conjugated goat anti-rabbit IgG antibody | Cell Signaling Technology | 7074 |
| α-actinin antibody | Proteintech | 11313-2-AP |
| Vimentin antibody | Proteintech | 10366-1-AP |
| β-MyHC antibody | Proteintech | 22280-1-AP |
| ANP antibody | Proteintech | 27426-1-AP |
| COL-1 antibody | Proteintech | 14695-1-AP |
| TGF-β1 antibody | Proteintech | 21898-1-AP |
| Myc tag antibody | Proteintech | 16286-1-AP |
| DYKDDDDK tag antibody | Proteintech | 66008-4-Ig |
| HA tag antibody | Proteintech | 51064-2-AP |
| GFP tag antibody | Proteintech | 50430-2-AP |
| FITC-conjugated wheat-germ agglutinin (WGA-FTIC) | Gene Tex | GTX01502 |
| OTUD1 antibody | Bioss | bs-17563R |
| H&E kit | Solarbio Life Sciences | G1120 |
| Picro Sirius Red stain | Solarbio Life Sciences | S8060 |
| mouse IL-6 uncoated ELISA Kit | ebioscience | 88-7064-77 |
| mouse TNF-α uncoated ELISA Kit | ebioscience | 88-7324-88 |
| RNAiso Plus | Takara | 9109 |
| PrimeScript™ RT reagent kit | Takara | RR037A |
| SYBR Green reagent kit | Vazyme | R223-00 |
| RIPA buffer | Beyotime Biotech | P0013C |
| Bradford assay | Bio-Rad | 5000205 |
| enhanced chemiluminescence | Bio-Rad | 1705062 |
| osmotic pump | Alzet MODEL | 1002 |

**Supplementary Table S2. Primer sequences for qPCR.**

| Gene | Species | Squence |
| --- | --- | --- |
| *Card3* | Mouse | AAATCATCCCCCACAGGAG  GGTCCAGGAGAACCAGTGTT |
| *Card4* | Mouse | TTTAAGGGTGAAGCCAAAGG  GGCAGACAAATCAGGATTCAG |
| *Card5* | Mouse | GAGCAGCTGCAAACGACTAA  GTCCACAAAGTGTCCTGTTCTG |
| *Card6* | Mouse | TTTCCTCCGGTGTTTGTCTAATG GTTCACCCCCACAGTCTCTTC |
| *Card9* | Mouse | ACTATGAGAATGACGACGAGTGC  GATCCGGGAGGGGTCAATG |
| *Card10* | Mouse | TCAGCACCTACCGTTTCCC  CCTCATCAAGGATCATGGAGC |
| *Card11* | Mouse | TCTCCAGAGCGAGTTTCTTCTT  TGTTTTCTGACCGGCTGAC |
| *Card12* | Mouse | TGATCTCCAAGAGATGAAGTTGG  GATCAAATTGTGAAGATTCTGTGC |
| *Card14* | Mouse | CAGCCGCATGAAACGTGAG  TCCTTCTCTCTGAGTGCGTTG |
| *Card15* | Mouse | CAGGTCTCCGAGAGGGTACTG GCTACGGATGAGCCAAATGAAG |
| *Myh7* | Mouse | ACTGTCAACACTAAGAGGGTCA TTGGATGATTTGATCTTCCAGGG |
| *Nppa* | Mouse | AAGAACCTGCTAGACCACCTGGAG TGCTTCCTCAGTCTGCTCACTCAG |
| *Col1a1* | Mouse | TGGCCTTGGAGGAAACTTTG  CTTGGAAACCTTGTGGACCAG |
| *Tgfb1* | Mouse | CTCCCGTGGCTTCTAGTGC  GCCTTAGTTTGGACAGGATCTG |
| *Il6* | Mouse | GAGGATACCACTCCCAACAGACC  AAGTGCATCATCGTTGTTCATACA |
| *Tnf* | Mouse | TGATCCGCGACGTGGAA  ACCGCCTGGAGTTCTGGAA |
| *Cxcr2* | Mouse | ATGCCCTCTATTCTGCCAGAT  GTGCTCCGGTTGTATAAGATGAC |
| *Cxcl1* | Mouse | CTGGGATTCACCTCAAGAACATC  CAGGGTCAAGGCAAGCCTC |
| *Clec7a* | Mouse | GGGTGCCCTAGGAGGTTTTT  TGCTGATCCATCCTCCCAGA |
| *Clec6a* | Mouse | AAGCGGAGCAGAATTTCATCA  CCATTTGCCATTACCTTGTGGA |
| *Clec4e* | Mouse | AGTGCTCTCCTGGACGATAG  CCTGATGCCTCACTGTAGCAG |
| *Otud1* | Mouse | AGAGGCAGGACAAGTACCTGA  CCCGTACACAGTCTTGCTGAC |
| *Usp15* | Mouse | CCGTGGATGAAAACCTGAGTAG  TTCTCTTAGGCAGACAGGGATAA |
| *Trim62* | Mouse | TGCGAGCACTACTTCTGCC  CTTGACCTTGTCGTGAGCC |
| *Actb* | Mouse | CCGTGAAAAGATGACCCAGA  TACGACCAGAGGCATACAG |
